# Ni-Xides (B, S, and P) for Alkaline OER: Shedding Light on Reconstruction Processes and Interplay with Incidental Fe Impurities as Synergistic Activity Drivers

**DOI:** 10.1021/acsaem.3c03114

**Published:** 2024-02-08

**Authors:** Sayed Mahmoud El-Refaei, David Llorens Rauret, Alba G. Manjón, Ioannis Spanos, Aleksandar Zeradjanin, Stefan Dieckhöfer, Jordi Arbiol, Wolfgang Schuhmann, Justus Masa

**Affiliations:** † Analytical Chemistry, Center for Electrochemical Sciences (CES), Faculty of Chemistry and Biochemistry, Ruhr University Bochum, Universitätsstr. 150, D-44780 Bochum, Germany; ‡ Catalan Institute of Nanoscience and Nanotechnology (ICN2), CSIC and BIST, Campus UAB, Bellaterra, 08193 Barcelona, Catalonia Spain; § ICREA, Pg. Lluís Companys 23, 08010 Barcelona, Catalonia, Spain; # Max-Planck-Institut für Chemische Energiekonversion, Stiftstraße 34-36, 45470 Mülheim an der Ruhr, Germany

**Keywords:** Oxygen Evolution Reaction, Reconstruction, Nickel Hydroxide, Nickel Phosphide, Nickel Sulfide, Nickel Boride

## Abstract

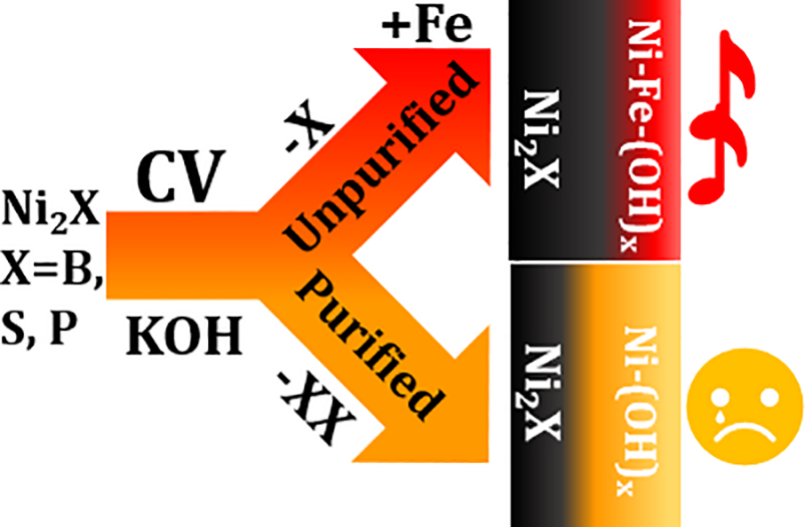

Ni-Xides (X = B, P, or S) exhibit intriguing properties that have endeared them for electrocatalytic water splitting. However, the role of B, P, and S, among others, in tailoring the catalytic performance of the Ni-Xides remains vaguely understood, especially if they are studied in unpurified KOH (Un-KOH) because of the renowned impact of incidental Fe impurities. Therefore, decoupling the effect induced by Fe impurities from inherent material reconstruction processes necessitates investigation of the materials in purified KOH solutions (P-KOH). Herein, studies of the OER on Ni_2_B, Ni_2_P, and Ni_3_S_2_ in P-KOH and Un-KOH coupled with *in situ* Raman spectroscopy, *ex situ* post-electrocatalysis, and online dissolution studies by ICP-OES are used to unveil the distinctive role of Ni-Xide reconstruction and the role of Fe impurities and their interplay on the electrocatalytic behavior of the three Ni-Xide precatalysts during the OER. There was essentially no difference in the OER activity and the electrochemical Ni^2+^/Ni^3+^ redox activation fingerprints of the three precatalysts via cyclic voltammetry in P-KOH, whereas their OER activity was considerably higher in Un-KOH with marked differences in the intrinsic activity and evolution of the Ni^2+^/Ni^3+^ fingerprint redox peaks. Thus, in the absence of Fe in the electrolyte (P-KOH), neither the nature of the guest element (B, P, and S) nor the underlying reconstruction processes are decisive activity drivers. This underscores the crucial role played by incidental Fe impurities on the OER activity of Ni-Xide precatalysts, which until now has been overlooked. *In situ* Raman spectroscopy revealed that the nickel hydroxide derived from Ni_2_B exhibits higher disorder than in the case of Ni_2_P and Ni_3_S_2_, both exhibiting a similar degree of disorder. The guest elements thus influence the degree of disorder of the formed nickel oxyhydroxides, which through their synergistic interaction with incidental Fe impurities concertedly realize high OER performance.

## Introduction

Electrochemical water splitting (EWS) driven by green electricity (e.g., solar and wind) to produce hydrogen is envisioned as the heart of the hydrogen economy.^1,2^ Yet, for cost-effective EWS to be realized, efficient electrocatalysts for the much more sluggish oxygen evolution reaction (OER), the counter-reaction during the hydrogen evolution reaction (HER), are required.^2^ Noble-metal-based catalysts (e.g., IrOx and RuOx) are the OER benchmark catalysts in acidic EWS; however, their high cost and scarcity urge the search for earth-abundant but efficient alternative electrocatalysts possessing high thermodynamic stability in acidic environments.^2,3^ In this regard, the mature alkaline EWS, despite its relatively lower efficiency compared to its acidic EWS counterpart, is a good compromise to mitigate the high cost of noble-metal-based electrocatalysts since it enables utilizing earth-abundant 3d transition-metal-based catalysts (e.g., Ni, Co, Fe, etc.).^2,3^ Ni-based catalysts are the most used in commercial electrolyzers owing to their high activity and stability. Driven by this commercial success, there is widespread research aiming to unravel the reasons behind the outstanding performance of nickel-based materials as OER electrocatalysts in alkaline electrolytes and introduce new strategies to maximize both their activity and stability.^4^

In alkaline electrolytes, the surfaces of Ni-based materials, including pure nickel, undergo transformation to nickel hydroxide Ni(OH)_2_ under an open-circuit potential. Ni(OH)_2_ occurs in two polymorphs, α-Ni(OH)_2_ and β-Ni(OH)_2_, which undergo oxidation to the corresponding oxyhydroxides, γ-NiOOH and β-NiOOH, respectively, under the oxidative OER potentials according to the well-known Bode scheme.^5^ Accordingly, the γ/β-NiOOH phases are believed to be the actual active form catalysts, of which γ-NiOOH is the most active one.^6^ Alloying Ni with other transition metals like Fe and Co is one of the adopted strategies to improve their performance.^7,8^ Among them, NiFe-hydroxide has attracted great attention in recent years as one of the best-known active nonplatinum group OER catalysts.^8-11^ Even though this system has been subjected to extensive investigations, definitive conclusions about the nature of the real active sites remain elusive.^12^ Some reports have proposed based on results from X-ray absorption spectroscopy (XAS) coupled with DFT studies that in the NiFe-hydroxide system, Fe and not Ni is the real active site while Ni(OH)_2_ serves as the host matrix.^13^ However, other studies have reported that Fe alloying inside Ni(OH)_2_/NiOOH induces electronic modulation, thus enhancing the performance of Ni active sites.^9,10^ There is a general consensus that alloying Ni with up to 25–30 at. % of Fe gives the most optimal OER performance, otherwise, an activity/stability drop is unavoidable.^9^ Extensive research on understanding the activity of the Ni_*x*_Fe_1–*x*_-(OH)_*y*_ system has led to the conclusion that the presence of a trace amount of Fe in KOH/NaOH electrolyte solutions plays a decisive role for the activity/stability of Ni(OH)_2_ catalysts.^9^ At subppm concentrations, it is evident that Fe can incorporate inside the accessible Ni(OH)_2_ structure during electrochemical studies ending up with Ni_*x*_Fe_1–*x*_-(OH)_*y*_ rather than the initial Ni(OH)_2_ structure, thus unraveling an important reason behind the apparent high activity/stability of Ni(OH)_2_.^9^ Moreover, these studies have also shown that incidental Fe incorporation can result in an atomic composition resembling that of the originally designed Ni_*x*_Fe_1–*x*_-(OH)_*y*_ system, where Fe at. % can reach 25–30% depending on the electrochemical assessment protocol.^14,15^ These findings motivated increased interest to devise new design strategies for efficient Ni-based OER catalysts. For instance, instead of synthesizing Ni_*x*_Fe_1–*x*_-(OH)_*y*_ catalysts via complicated sol–gel, hydrothermal, or thermal techniques, among others, where elemental segregation is possible, Fe could be introduced later via electrochemical treatment of monometallic Ni-based catalysts in predesigned alkaline solutions containing Fe to attain high activity/stability performance.^16,17^

Ni alloyed with nonmetals (e.g., C, B, P, S, Se, etc.), referred to as Ni-Xides (X = C, B, P, S, Se, etc.), has drawn considerable attention in recent years as a promising strategy to produce viable OER catalysts.^18^ Due to thermodynamic instability in alkaline solution and under OER conditions, Ni-Xides are prone to undergo transformation to the corresponding hydroxide form.^18−24^ This transformation process, also known as “reconstruction” in the literature, might be either deep, resulting in a complete transformation of the Ni-Xide to hydroxide, or a partial surface reconstruction, ending up with a core@shell (Ni-Xide@Ni(OH)_2_) structure depending on the particle sizes and applied electrochemical activation protocols. Ni-Xides are thus more appropriately referred to as precatalysts rather than catalysts.^20,22,25^ Even though there is some controversy regarding the extent of the reconstruction, it is universally believed to be the main reason behind the prominent activity exhibited by Ni-Xides. More specifically, the hydroxides formed from the reconstruction processes possess higher surface areas and more abundant active sites as well as defective structures compared to pristine bulk hydroxide catalysts.^20^ Additionally, the core–shell morphology guarantees fast electron transport across the catalyst layer, which in turn facilitates charge transfer at the interface.^20^ Despite the immense amount of work emphasizing the reconstruction of Ni-Xides as an evident strategy to drive efficient OER electrocatalysis, the impact of incidental Fe that might incorporate in the generated Ni_*x*_(OH)_*y*_ during the electrochemical activity/stability studies has been mostly overlooked.^15,26^ This raises questions regarding the validity of the role of the reconstruction process as the main activity driver and obscures a definitive understanding of the exact reasons for the prominent activity of Ni-Xide catalysts. For example, Hu et al. reported that nickel sulfides have OER activity in the order of Ni_3_S_2_ > NiS > NiS_2_ in 1.0 M KOH that was attributed to the higher metallic character in the sulfur-deficient phases.^27^ However, the KOH was used without purification, pointing to the possibility of Fe incorporation being a potential additional reason or even the main reason, if we bear in mind that pure Ni(OH)_2_ is a less active electrocatalyst in Fe-free KOH according to the work of Boettcher et al.^28^ Incidental Fe has also been proven to positively bolster the stability of Ni-based catalysts in alkaline media. For instance, using online inductively coupled plasma optical emission spectroscopy (ICP-OES), Spanos et al. observed that Ni–Co_3_O_4_ exhibits bad stability in 1.0 M Fe-free KOH attributed to continuous corrosion of Ni and Co from the catalyst surface; however, in unpurified KOH, besides higher activity, the stability was also enhanced and the corrosion rate greatly diminished.^29^ In this context, the good stability reported for Ni-Xides in the literature is expected to be partially due to the impact of incidental Fe in unpurified KOH solutions. Mullins et al. reported two successive studies on the impact of incidental Fe impurities on the activity and stability of two different Ni-Xide precatalysts, namely, Ni_3_N and NiSe.^14,15^ Their experimental findings revealed that the prominent activity of the derived hydroxide catalysts is mainly attributed to Fe incorporation, which modulates the electronic structure of the host Ni(OH)_2_ matrix. For instance, Ni_3_N/Ni afforded 20 mA cm^–2^ at 325 and 482 mV in unpurified and purified 1.0 M KOH, respectively.^14,30^ The value in unpurified KOH matches that reported for the same materials in the previous literature (365 ± 24 mV at 20 mA cm^–2^), further emphasizing that Fe played a decisive role that must be unraveled.^14^ A recently adopted approach to impart high electrocatalytic performance to Ni-Xide precatalysts is to simultaneously incorporate different nonmetals in the same structure.^31^ For example, doping metallic Ni_3_N with S and P proved efficient in enhancing the performance relative to Ni_3_N/P as reflected in a lower OER overpotential, lower charge transfer resistance, and higher electrochemical active surface area (ECSA).^32^*In situ* Raman spectroscopy revealed initial irreversible reconstruction of the surface layer, leading to the formation of a reversible OER efficient α-Ni(OH)_2_/γ-NiOOH redox pair. Likewise, incorporating several chalcogens in the same structure was shown to exhibit better performance than the mono Ni-Xides, as in the case for Ni_3_Se_4_ doped with S or vice versa.^31,33^ However, in these studies, Fe incorporation was not considered, which might point to possible differences in the extent and mechanism of Fe incorporation in the Ni-Xides or to a different reconstruction mechanism.

In the current study, rather than focusing on only one example of Ni-Xides to derive the impact of reconstruction processes and the role of Fe impurities on the electrocatalytic performance, three kinds of Ni-Xides, namely, Ni_2_B, Ni_3_S_2_, and Ni_2_P, were selected. This selection would enable us to derive generic conclusions about reconstruction processes, the role of incidental Fe impurities in the electrolyte, and their interplay as synergistic activity drivers, as well as how to employ this insight into designing more efficient Ni-Xide precatalysts. The overarching aim was to address three main points: first, (I) since the three Ni-Xides share a close Ni:X ratio (2:1), compare their activity in the same manner to reveal which of them is most promising is justifiable, keeping in mind that X possesses different atomic radii and electronegativity; second, (II) investigate the impact of incidental Fe on the activity of the derived NiOOH and whether the initial Ni-Xide precatalysts play a decisive role or not; and third, (III) highlight the impact of the presence and absence of Fe in the electrolyte (KOH) on the degree or extent of the reconstruction process and thereby understand whether the nature of the initial Ni-Xide precatalyst is relevant or not.

## Results and Discussion

**Figure 1:**
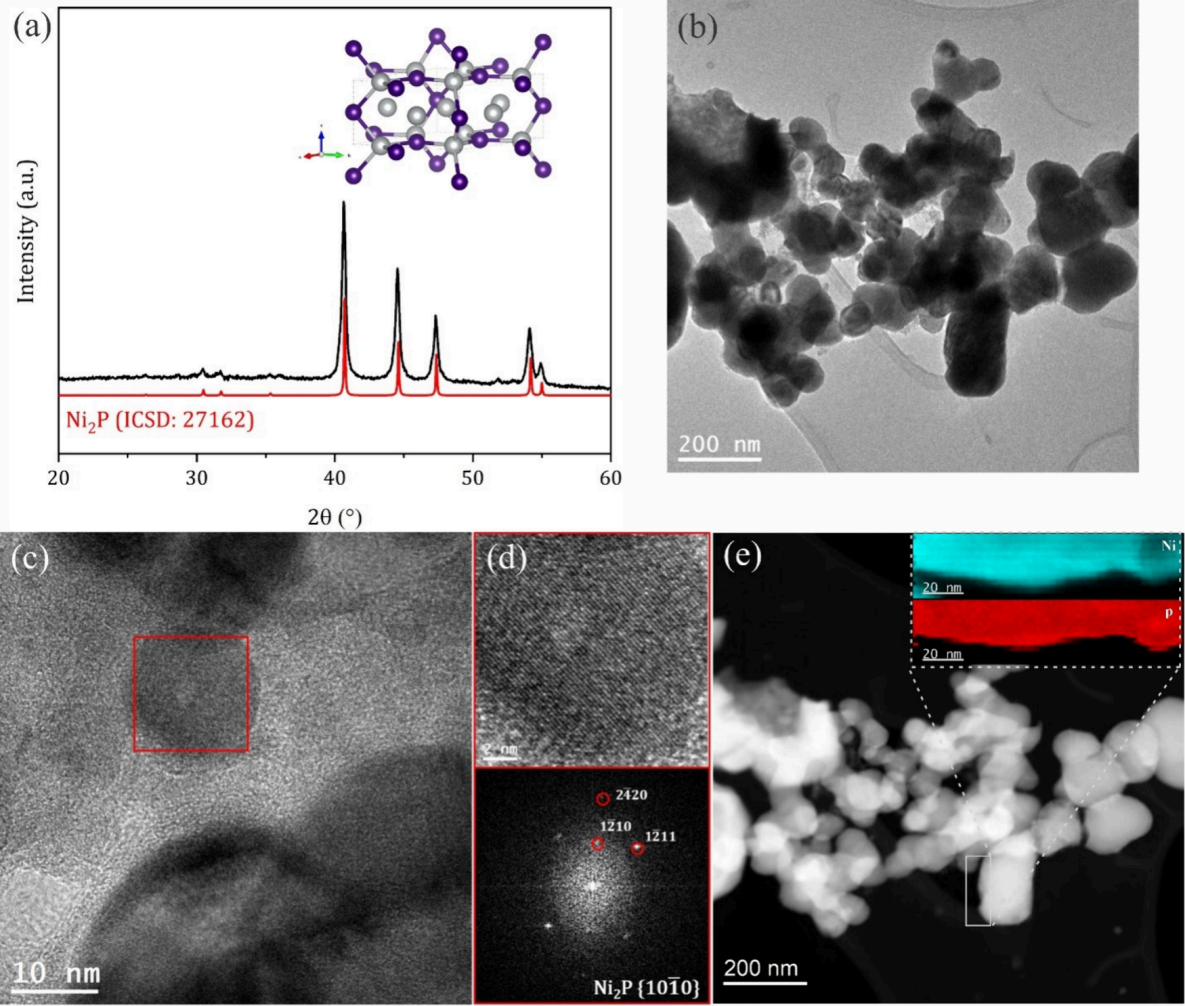
(a) XRD pattern for Ni_2_P, (b) TEM, and (c and d) HR-TEM with corresponding indexed FFT. (e) HAADF-STEM image with corresponding EELS mapping.

Ni_2_P,^34^ Ni_3_S_2_,^35^ and Ni_2_B^36^ were synthesized according to the literature as introduced in the Experimental Section. The synthesized materials were characterized first by using powder X-ray diffraction (XRD) and transmission electron microscopy (TEM) to confirm their structure and crystallinity. Figure 1a shows the XRD pattern of an as-synthesized Ni_2_P crystallized in the hexagonal Ni_2_P structure without any discernible impurity peaks. TEM reveals an irregular granular morphology (Figure 1b). High-resolution TEM and corresponding fast Fourier transform (FFT) analysis in Figures 1c and 1d reveal a well-crystallized Ni_2_P without defects or impurities, corroborating the XRD results. The high-angle annular dark field scanning TEM (HAADF-STEM) image (Figure 1e) shows that Ni_2_P has a compact structure with the corresponding electron energy loss spectroscopy (EELS) composition mapping confirming the homogeneous distribution of Ni and P with a ratio of 2:1 matching the Ni_2_P crystal structure across the entire mapped area. Corresponding analyses of Ni_3_S_2_ and Ni_2_B are shown in Figures S1 and S2. XRD and TEM/HR-TEM in Figure S1 show that nickel sulfide crystallized in the trigonal Ni_3_S_2_ crystal structure (ICSD 23114) with minor diffraction peaks belonging to NiS. The nickel boride XRD pattern and TEM/HR-TEM corroborate the successful synthesis of Ni_2_B that crystallized in the tetragonal Ni_2_B structure (ICSD 75792). The tiny diffraction peaks are attributed to boron-deficient Ni_3_B, which cannot be easily avoided during the solid-state reaction. The corresponding XRD patterns and TEM analysis proved that the three targeted materials were synthesized successfully, albeit with minute impurities that are not expected to significantly affect the electrochemical study. Additionally, the three materials had comparable crystallite sizes of 18.74, 24.89, and 21.84 nm for Ni_2_B, Ni_3_S_2_, and Ni_2_P, respectively, as derived from the Scherrer equation.

**Figure 2:**
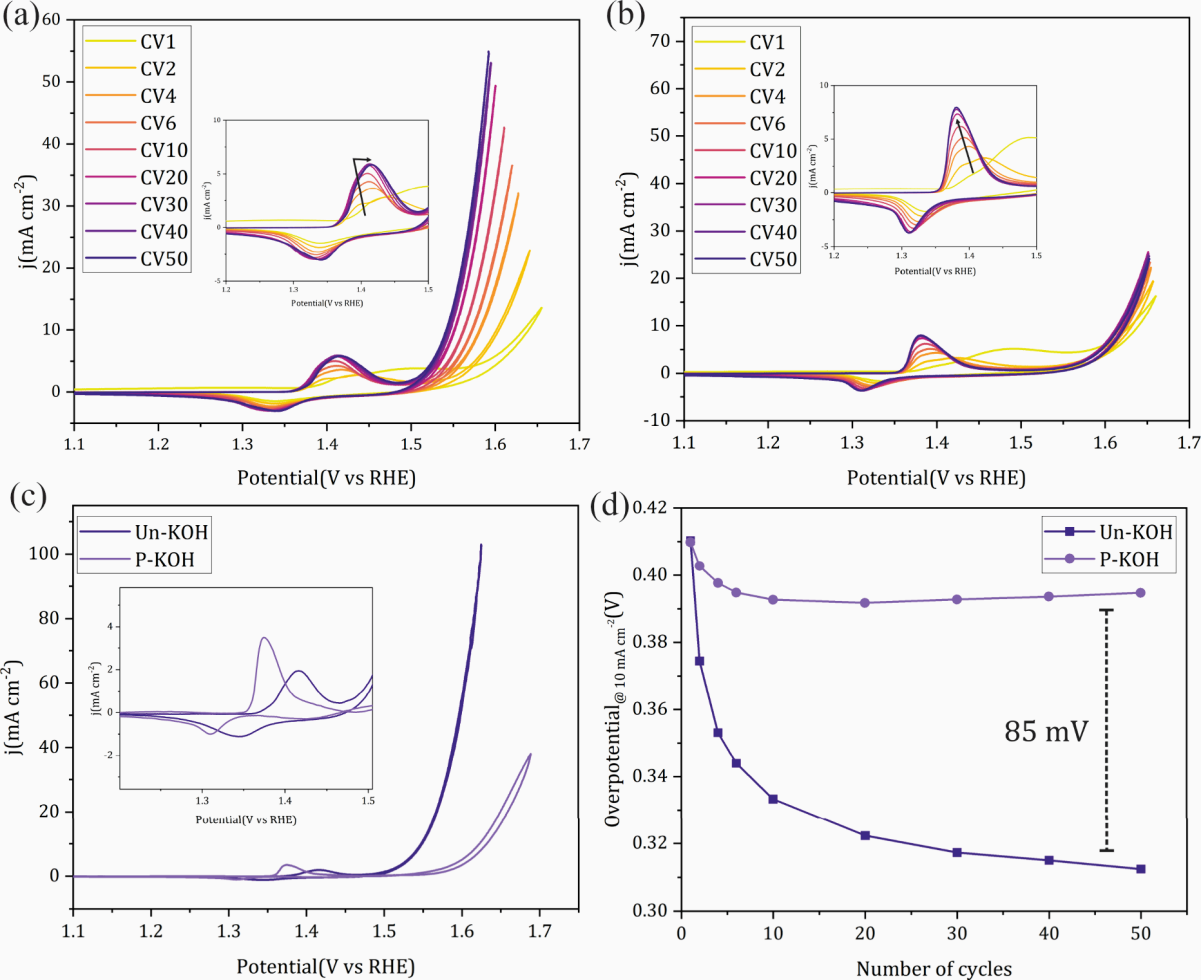
Fifty continuous CVs for the activation of Ni_2_P recorded at a scan rate of 20 mV s^–1^ in (a) 1.0 M Un-KOH and (b) 1.0 M P-KOH. (c) CV recorded at 5 mV s^–1^, after the activation process (a and b), showing a comparison of the OER performance of Ni_2_P in Un-KOH and P-KOH; inset is the magnification of Ni^2+/3+^ redox peaks. (d) The corresponding overpotentials at 10 mA cm^–2^ derived from a and b.

The electrochemical evaluation was conducted in 1.0 M KOH, either unpurified (Un-KOH) or purified (P-KOH), in a three-electrode cell employing glassy carbon (0.196 cm^–2^) as the working electrode with catalyst loadings of 31 μg cm^–2^. As nickel phosphides are reported as one of the most efficient and well-studied OER catalysts in the literature, Ni_2_P is introduced herein as the representative system, while Ni_2_B and Ni_3_S_2_ are mentioned for the purpose of comparison. Cyclic voltammetry (CV) was adopted as the activation protocol to study the reconstruction process as this technique enables direct observation of redox changes on the catalyst. Figure 2a shows 50 consecutive CVs of Ni_2_P in 1.0 M Un-KOH at 20 mV s^–1^. As expected, the first CV exhibits poor OER performance as well as the weakly resolved and delayed appearance of Ni^2+^/Ni^3+^ redox peaks, pointing to the fact that a small fraction of the Ni_2_P surface is reconstructed to the corresponding Ni_*x*_(OH)_*y*_ upon contact with the KOH solution. On successive cycling up to 30 CVs, the Ni^2+^/Ni^3+^ redox peaks became better resolved and gradually shifted to lower potentials with concurrent enhancement of the OER performance. Afterward, the Ni^2+^/Ni^3+^ redox potential started to shift to a higher potential with concurrent saturation of the OER activity. To have a closer look at the change of the OER performance with the consecutive CVs, the overpotential at 10 mA cm^–2^ as a figure of merit is extracted from the IR-corrected data and plotted against CV number during cycling. As can be seen in the first CV of Figure 1d, an overpotential of 410 mV was required to attain 10 mA cm^–2^, which dropped significantly with cycling, reaching a steady value of about 310 mV after 50 CVs. Ni_3_S_2_ exhibited similar redox changes and potential shifts as well as an enhanced OER (Figures S3a and S3d). The same was true for Ni_2_B (Figure S4); however, the overpotential drop at 10 mA cm^–2^ was not as significant as in the case of Ni_2_P and Ni_3_S_2_ and the Ni^2+/3+^ redox peak only shifted positively with cycling. The discrepancy between the behavior of Ni_3_S_2_/Ni_2_P and Ni_2_B could be partially attributed to the washing process during the synthesis of Ni_2_B, where hot water was employed to remove the KCl/LiCl salt mixture, which could induce prior oxidation to Ni-oxide/hydroxide surface species. These results are in accordance with the literature for the same class of materials.^37,38^

Significant changes can be observed during the activation of Ni_2_P in P-KOH (Figure 2b). First, the redox charge capacity of Ni^2+^/Ni^3+^ is clearly larger, more resolved and slightly shifted to lower potentials, indicating possibly more pronounced surface reconstruction leading to a thicker hydroxide layer and easier redox switching compared to the case of Un-KOH (Figure 2b, inset). Second, the OER with cycling was minimal, in sharp contrast to that in Un-KOH even though the voltammetric behavior indicates more facile kinetics of Ni_*x*_(OH)_*y*_ formation in the former, thus highlighting the fact that other factors contribute, at least in part, to the higher apparent activity of the catalyst in Un-KOH. Figure 2d compares the changes in the overpotential values with those obtained by cycling at 10 mA cm^–2^ in both KOH solutions. Even though the overpotential generally decreased with cycling, initially, the activity changes in Un-KOH are markedly larger, pointing out that the reconstruction process, which was more pronounced in P-KOH, is not the main driver for the apparent overpotential drop in Un-KOH, where the overpotential difference at 10 mA cm^–2^ after 50 CVs of continuous potential cycling was 85 mV. The less pronounced decrease in the overpotential in the case of P-KOH could be attributed to the thicker hydroxide shell possessing lower electronic conductivity and in turn lower overall OER activity. Conversely, in Un-KOH (Figure 2C), the lower charge capacity could mean a thinner hydroxide shell and that the presence of Fe impurities in the electrolyte led to increased conductivity of the catalytic film and modulation of its electronic structure. To better imagine the effect of electrochemical reconstruction in both KOH media on the OER activity of *in situ*-derived Ni_*x*_(OH)_*y*_, CVs to higher potentials in the OER regime were measured at 5 mV s^–1^ and are shown in Figure 2c. The OER activities differ significantly, where in Un-KOH at 1.6 V vs RHE the catalyst reached a current density of 57 mA cm^–2^, about 11 times higher than that in the case of P-KOH (5 mA cm^–2^) at the same potential. By comparison of the Ni^2+^/Ni^3+^ redox features (Figure 2c, inset), discernible differences of the impact of cycling in the different KOH solutions on the electrochemical behavior of the derived catalyst can be observed. It can easily be noted that the charge capacity of the catalyst derived in P-KOH is higher and the potential of the Ni^2+/3+^ redox peaks is shifted to a lower potential relative to the case of Un-KOH. These observations point to possible Fe incorporation in the surface Ni_*x*_(OH)_*y*_ catalytic layer as already reported in the literature.^28^ Ni_2_B and Ni_3_S_2_ exhibit the same features of significant OER enhancement with CV cycling and lower redox charge capacity as well as a shift of the redox peaks toward higher potentials in Un-KOH (Figures S3 and S4) in relation to P-KOH. From these results of three different Ni-Xides that exhibit similar electrochemical behaviors in different KOH solutions, one can infer that the impact of the presence of incidental Fe was consistent with literature reports.^14,15,39^ Moreover, the reconstruction process is well recognized as an important prerequisite for the formation of Ni_*x*_(OH)_*y*_ species, including Ni(OH)_2_ and NiOOH, which in turn interact with the Fe in solution to form efficient active sites similar to or related to the ones reported for pristine Ni(OH)_2_ cycled in 1.0 M KOH containing incidental Fe, as highlighted in the previous literature.^15,26^

**Figure 3:**
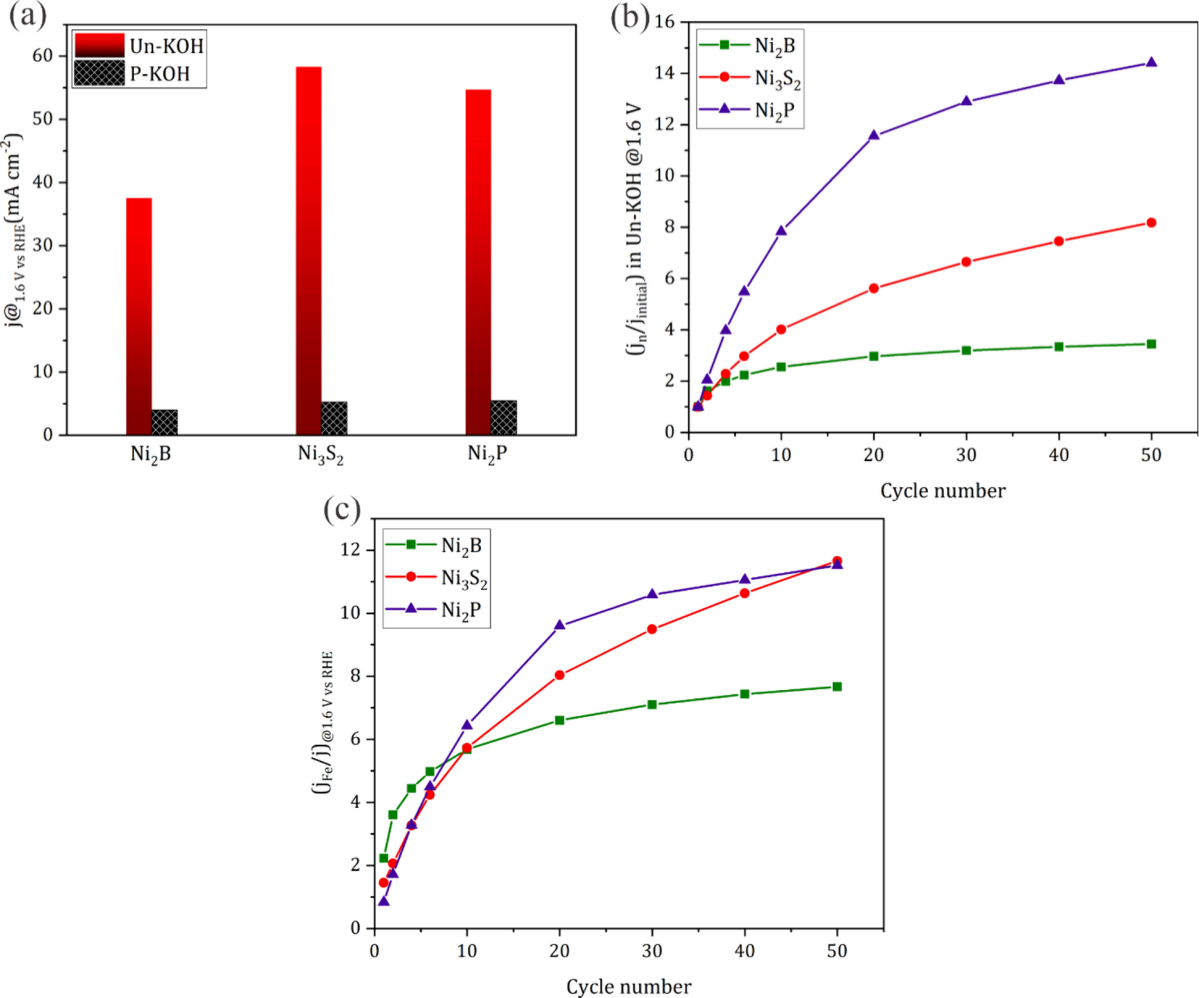
(a) Geometric current densities at 1.6 V vs RHE in 1.0 M Un-/P-KOH after 50 CVs. (b) Relative OER improvement at 1.6 V vs RHE for Ni_2_B, Ni_3_S_2_, and Ni_2_P in 1.0 M Un-KOH derived from the corresponding CVs. (c) Activity enhancement factor at 1.6 V vs RHE for Ni_2_B, Ni_3_S_2_, and Ni_2_P.

In the interest of understanding the possible role of Fe interplay during the reconstruction process and whether the nature of the initial Ni-Xide precatalyst is critical for both geometric and intrinsic activity, several factors have been extracted from the electrochemical data. As for geometric activity, the current density achieved by the different Ni-Xides at a fixed potential was calculated and compared (Figure 3a). In P-KOH, all three materials exhibited almost the same current density of around 5 mA cm^–2^ at 1.6 V vs RHE, suggesting that the initial structure is not primarily important. This was however not the case in Un-KOH, where besides the significant OER enhancement, markedly different current densities were observed. Ni_3_S_2_ and Ni_2_P both reached about the same value of 55 mA cm^–2^ at 1.6 V, whereas Ni_2_B reached only about 38 mA cm^–2^, accounting for 33% less activity relative to the case of Ni_2_P and Ni_3_S_2_. This considerable difference in activity between Ni_2_B and Ni_3_S_2_/Ni_2_P could be attributed to several reasons including different surface areas and in turn the number of exposed active sites, the distinct nature of active sites resulting in different reaction kinetics, different Fe incorporation levels, a different mechanism or extent of Fe interplay during the reconstruction process, or different leaching kinetics of B relative to P and S. In this regard, the relative OER improvement with cycling (*j*_*n*_/*j*_initial_), where *j*_*n*_ is the current density at 1.6 V vs RHE for CV number *n* and *j*_initial_ is the current density at the same potential for the first CV in 1.0 M Un-KOH, was calculated and presented in Figure 3b. As a general feature, the OER improved very fast, followed by a plateau depending on the nature of the sample. The OER activity of Ni_2_B doubled in only six CVs followed by a plateau where no further increase in the OER activity was observed. As for Ni_3_S_2_, the original current density increased four times after six CVs, double that of Ni_2_B, but the plateau was not reached as early as for the case of Ni_2_B, with the current continuing to increase slowly reaching an enhancement of eight times after 50 CVs. The activity improvement was much more rapid and significant in the case of Ni_2_P compared to Ni_3_S_2_ and Ni_2_B, where 8-fold enhancement was reached after only six CVs, reaching a remarkable 12 times enhancement after 50 CVs. The distinct differences in the activity enhancement of the three materials, where Ni_2_B showed the least improvement compared to Ni_3_S_2_ and Ni_2_P, could be attributed to different extents of X leaching and subsequent Fe uptake. Boron with a smaller atomic radius and lower electronegativity compared to S and P could have a lower leaching tendency, resulting in a more compact hydroxide layer with comparatively lower electrolyte permeability and in turn lower surface area and exposed active sites. These factors would lead to a faster saturation of the produced hydroxide with Fe resulting in no further increase in the activity. In contrast, P and S possessing bigger atomic radii could readily leach further with cycling, leaving behind a comparatively loose hydroxide layer that favors higher electrolyte permeability and thus more exposed active sites and subsequent Fe uptake. To further understand the distinctive activity enhancement between Ni_2_B and Ni_3_S_2_/Ni_2_P, the enhancement factor originally introduced by Markovic et al. was calculated and presented in Figure 3c. This factor, which is defined by EF = (*j*_Fe_/*j*_(non-Fe)_)_*n*_ at 1.6 V vs RHE, where *n* is the cycle number and *j*_Fe_ is normalized by the corresponding current for each cycle at the same potential but in the absence of Fe, gives a fairer picture of the Fe uptake effect at each cycle.^40^ Additionally, this is considered a more reliable way to directly compare the three Ni-Xides. The activity segmentation in the presence of Fe becomes clearer in Figure 3c, where Ni_3_S_2_ and Ni_2_P exhibit a similar trend different from that of Ni_2_B. Even though the enhancement factor in the case of Ni_2_P is faster than that of Ni_3_S_2_, the two materials reached the same current value after 50 CVs. One can infer from this point that phosphorus leaching apparently provides more active states relative to sulfur after the same number of CVs; however, with continuous cycling, the rate of phosphorus leaching starts to slow down while sulfur leaching continues finally reaching the same activated state as Ni_2_P, as will be highlighted later. Boron leaching in contrast could not render the same density of active states, affording only half of the enhancement factor of Ni_2_P and Ni_2_S_3_, and this could be attributed to the same reasons stated before, that is, a lower leaching tendency or formation of a more compact hydroxide with a smaller surface area.

**Figure 4:**
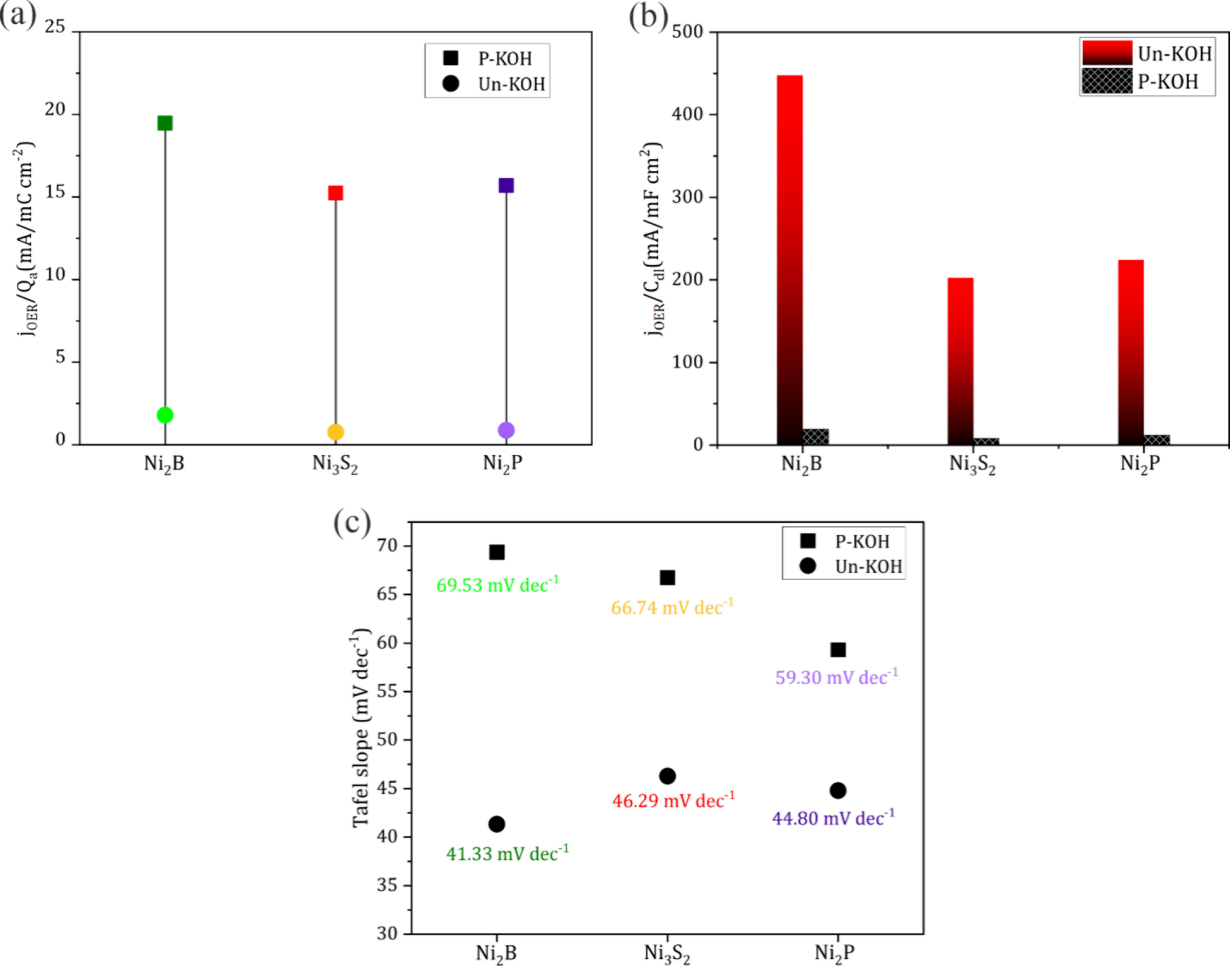
Current densities at 1.6 V vs RHE normalized by the corresponding *Q*_a_ (a) and normalized by the corresponding *C*_dl_ (b) in 1.0 M Un-/P-KOH after 50 CVs. (c) Tafel slopes for Ni_2_B, Ni_3_S_2_, and Ni_2_P in 1.0 M Un-/P-KOH.

The discussion in the previous section is built on the geometric current densities, which is helpful to understand the activity from an engineering point of view. To obtain deeper insight into the interplay between the reconstruction processes and the interaction of incidental Fe impurities, a comparison based on intrinsic activities would be more fruitful. To do so, the anodic charge capacity (*Q*_a_) of the Ni^2+^/Ni^3+^ redox wave, envisioned to correlate with the number of exposed active sites and KOH permeable layer, was extracted from the corresponding CVs measured at a slow scan rate of 5 mV s^–1^ to minimize the contribution of the charging current. Figure S5a shows that Ni_2_B had a lower *Q*_a_ compared to Ni_3_S_2_ and Ni_2_P, and the corresponding charge in Un-KOH is lower than in P-KOH. The current densities at 1.6 V vs RHE were normalized by *Q*_a_ aiming at comparing the absolute intrinsic activities. In P-KOH, even though the geometric current densities were the same, Ni_2_B exhibited a little higher value (1.84 mA/mC cm^2^) than Ni_2_P (0.93 mA/mC cm^2^) and Ni_3_S_2_ (1.08 mA/mC cm^2^) upon normalization with the corresponding charge, pointing to a possible lower extent of reconstruction in the case of boron leaching. In Un-KOH, the activity generally increased, consistent with the discussion before. Ni_2_B showed higher activity in relation to Ni_3_S_2_/Ni_2_P, indicating that the apparent higher activity presented by Ni_3_S_2_/Ni_2_P in Figure 3 could mainly be due to more exposed active sites rather than a difference in the intrinsic activity. Normalizing the current density by the double-layer capacitance (*C*_dl_) is another acceptable way to highlight the intrinsic activity of catalysts, especially if they are electrochemically treated in the same way. The corresponding *C*_dl_ values in Un-KOH and P-KOH are extracted from electrochemical impedance spectroscopy (EIS) measurements at 1.6 V vs RHE using a fitting model introduced in Figure S5b. Figure S5c reveals that Ni_2_B exhibited much lower *C*_dl_ than Ni_3_S_2_ and Ni_2_P in the different KOH solutions, further emphasizing the lower comparative surface area as stated before, which also aligns with the *Q*_a_ calculations. The results of the current normalized with *C*_dl_ are shown in Figure 4b, which further emphasizes that the hydroxides derived from the three Ni-Xides exhibit comparable intrinsic activities in P-KOH. However, the presence of Fe during the reconstruction process induces a different effect upon them. Ni_2_B showed higher intrinsic activity in relation to Ni_3_S_2_ and Ni_2_P that exhibited the same activity, which aligns with the enhancement factor between the two groups, thus indicating that the higher geometric activity exhibited by both Ni_3_S_2_/Ni_2_P (Figure 3a) is mainly attributed to the higher accessible density of active sites. To conclude whether only the accessible density of the active sites and not the inherent difference in their composition is the main reason, Tafel slopes were extracted from the CVs measured at a low scan rate of 5 mV s^–1^. Kinetic analysis confirmed the formation of the same nature of active sites in P-KOH for all three catalysts exhibited Tafel slopes in the same kinetic regime (69–60 mV dec^–1^), as shown in Figure 4c. In Un-KOH, the Tafel slopes were significantly lower but in the same kinetic regime and closer to each other compared to the case of P-KOH. This kinetic analysis together with the intrinsic activity presented in Figures 4a and 4b reveals that the presence of Fe in the KOH induces the formation of a new kind of active site that is the same regardless of the starting Ni-Xide and is envisioned to comprise a Ni–Fe hybrid oxo-hydroxide species.

**Figure 5:**
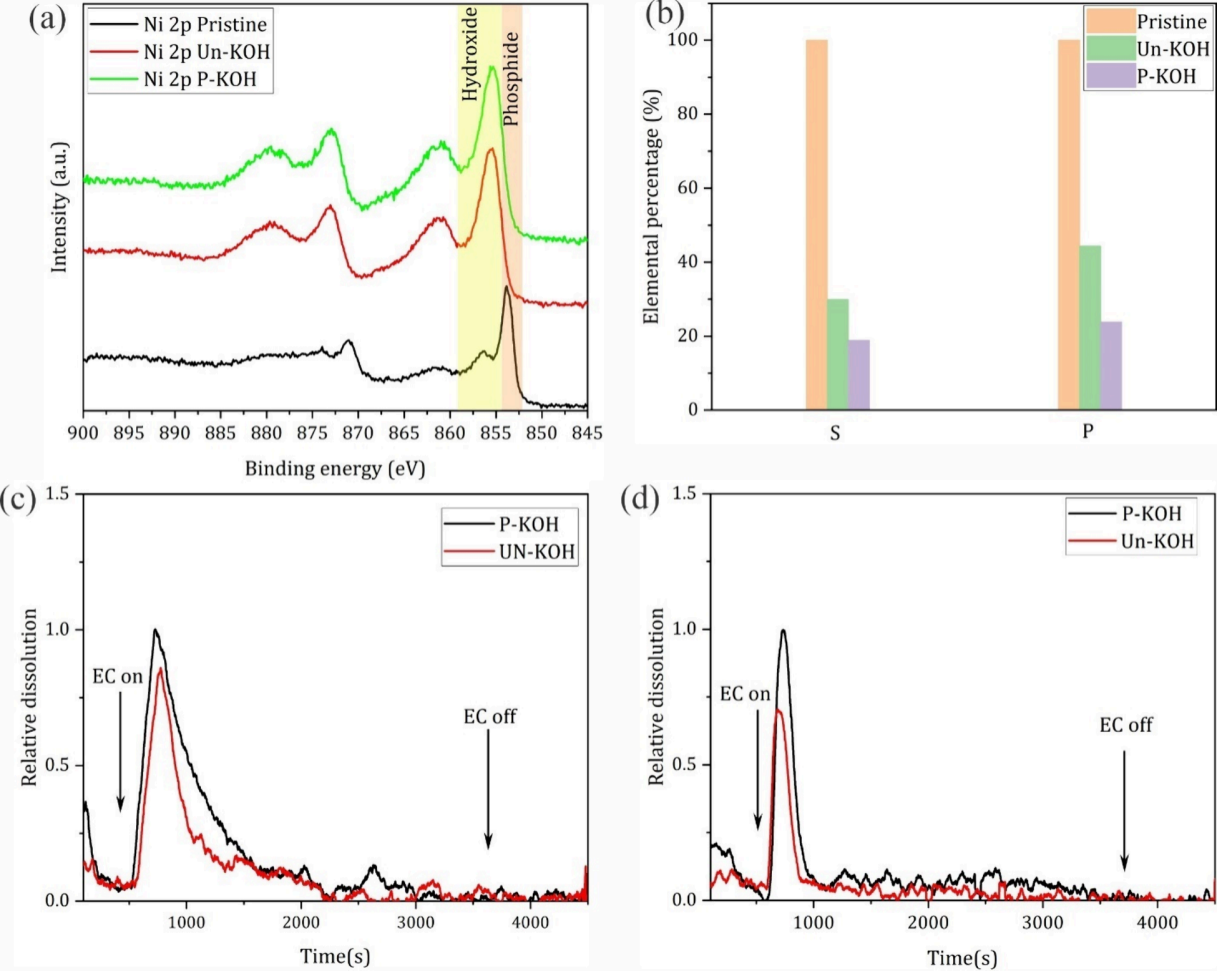
(a) XPS spectra of the Ni 2p core level of Ni_2_P for the pristine and reconstructed samples (after 50 CVs) in Un-KOH and P-KOH. (b) Elemental composition of residual P and S determined by EDS in samples of Ni_2_P and Ni_3_S_2_, respectively, after 50 activation CVs in Un-KOH and P-KOH. ICP-OES online analysis during 50 CVs of activation of Ni_2_P (c) and Ni_3_S_2_ (d) in Un-KOH and P-KOH.

**Figure 6:**
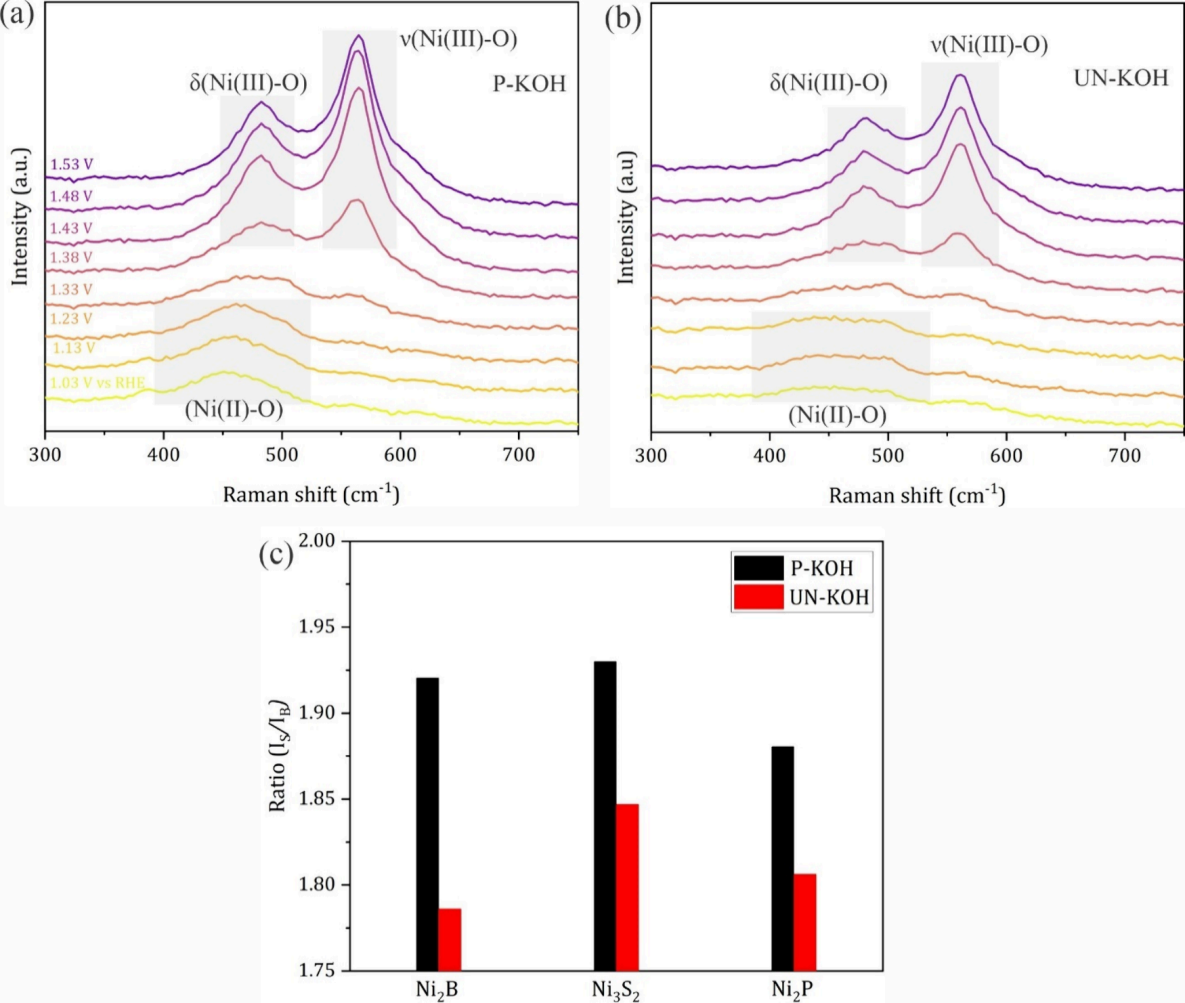
Raman spectra of activated Ni_2_P at different potentials in (a) P-KOH and (b) Un-KOH. (c) Intensity ratio of the ν_(Ni(III)-O)_ to δ_(Ni(III)-O)_ peaks at 1.53 V in P-KOH and Un-KOH.

To probe the surface chemical composition before and after the reconstruction step under different conditions, X-ray photoelectron spectroscopy (XPS) was employed. Figure 5(a) shows the Ni 2p spectra for Ni_2_P in P-KOH and Un-KOH in comparison to the pristine samples. The as-synthesized Ni_2_P exhibits a narrow Ni 2P_3/2_ peak at ca. 853.7 eV, attributed to Ni states in the phosphide structure. After the reconstruction in Un-KOH, the Ni 2p_3/2_ peak broadened and shifted to higher binding energy indicating the formation of oxidized Ni species, presumably surface hydroxides, induced by the thermodynamic instability of Ni_2_P in alkaline media. Interestingly, the Ni 2p_3/2_ signals of the generated hydroxide materials in P- and Un-KOH resemble each other in terms of both peak shape and position, pointing to the similarity of their chemical structure. In the case of Ni_3_S_2_ (Figure S6a), the pristine sample shows signal contributions of sulfides at relatively low binding energy and contributions from hydroxide species at higher binding energy induced by surface oxidation in air. After the reconstruction, the lower binding energy contribution of sulfides vanished while the one attributed to hydroxides at higher energy was enhanced. Notably, there was a resemblance between the features of the surface hydroxide structures generated in both P-KOH and Un-KOH. In the case of Ni_2_B, the Ni 2p region spectrum of the pristine sample suggests the presence of a hydroxide rather than boride structure, indicating the presence of a native hydroxide shell on the boride structure induced by the washing step in hot water to get rid of the residual salts from the synthesis process, as already explained. After electrochemical treatment in P-KOH and Un-KOH, the region spectra did not change visibly, essentially matching each other to a large extent (Figure 6b). At this point it is important to mention that the XPS measurements were conducted for the reconstructed samples *ex situ* where the samples were exposed to air that might have induced some structural changes. Even though the hydroxide materials generated in both P-KOH and Un-KOH from the different starting Ni-Xide exhibit similar XPS features, the *ex situ* analysis is not enough to deduce the possible reasons behind the different electrochemical behaviors of the generated hydroxides and especially to explain the differences between hydroxides generated from different Ni-Xide sources. To have a general overview regarding the leaching of the guest elements and the role that the composition of the KOH electrolyte might play, the working electrodes were examined with energy dispersive X-ray spectroscopy (EDS) in SEM mode after activation in P-KOH and Un-KOH for Ni_2_P and Ni_3_S_2_ only, as B is not accessible due to its poor detection by EDS and/or convolution of its signal with that of carbon. Figures S7 and S8 show EDS mapping of the electrodes after treatment in P-KOH and Un-KOH, where the percentages of the remaining P and S are extracted and compared in Figure 5b. It is evident from the percentages of residual P and S that the extent of leaching in P-KOH is higher than that in Un-KOH even though the electrodes were subjected to the same number of CV cycles. This aligns with the aforementioned different electrochemical behaviors regarding the redox charge capacity of the Ni^2+^/Ni^3+^ peaks, where in P-KOH the peaks are more pronounced pointing to a deeper reconstruction, suggesting comparatively higher leaching of the respective guest elements. In this regard, online ICP-OES analysis was used to confirm the extent of leaching of the guest elements in P-KOH relative to Un-KOH. Figures 5c and 5d show the online ICP-OES data collected during 50 CVs. Coupling of the ICP-OES with the electrochemical cell was achieved by connecting the outlet tubing of our homemade flow cell to the inlet of ICP-OES.^29^ At the beginning of the experiments (the first 500 s), a little rise of both P and S signals was observed due to possible leaching and thus reconstruction at the open-circuit potential. Upon application of potential cycling (EC on), the P and S signals increased significantly due to further potential-induced leaching. The signals peaked and dropped again, reaching small values within different time scales depending on the leaching element. In the case of Ni_2_P, the major dissolution process spanned around 2000 s, while it spanned a shorter time of around 500 s in the case of Ni_3_S_2_. After the peak time, the dissolution continued yet with a small rate as the signal became closer to the baseline even though the CV experiment was still running. This might be attributed to the near complete dissolution of the guest elements within this time scale (deep reconstruction) or the partial dissolution induced surface passivation rendering further reconstruction hard (core–shell or heterostructure). The EDS analysis mentioned here before emphasizes the surface passivation possibility as a reason for the small signal of P and S with continuous CVs. The difference between Ni_2_P and Ni_3_S_2_ in the time-dissolution dependency under the same reconstruction conditions (CVs) points to possible different leaching mechanisms that could be employed to smartly engineer the surface properties and control the hydroxide thickness. Noticeably, the relative dissolution of P and S in P-KOH is higher than that in Un-KOH under the same reaction conditions, inferred from the noticeable bigger area under the peak within the same time scale. These observations further corroborate the EDS mapping results as well as the electrochemical behavior in P-KOH, that is, the absence of Fe induces more intense reconstruction and increased exposure of Ni surface sites. However, the OER catalytic activity in P-KOH is much worse than that in Un-KOH, underscoring the importance of Fe in the apparent catalytic activities of reported Ni-Xides. TEM/HR-TEM was applied to elaborate the surface changes of the catalysts activated under the different conditions. Figure S9 shows that the bulk structure of the Ni_2_P-derived catalyst was still Ni_2_P as inferred from the spacing and relative angle observed on the lattice fringes and the parallel indexation of FFT. However, from a closer look at the HR-TEM, one can infer that the evolution of surface amorphization (hydroxide formation) is more pronounced in P-KOH than Un-KOH, as evidenced by the defective lattice fringes. These observations further point to the more pronounced reconstruction in P-KOH relative to Un-KOH, thus aligning with the redox charge capacity, SEM-EDS, and ICP-OES analysis. Importantly, EDS mapping of the spent catalyst revealed *in situ* Fe absorption on the hydroxide layer. Figures S10 and S11 reveal that after 50 CVs in Un-KOH, the guest elements (P and S) still exist in the bulk structure, affirming the HR-TEM analysis. Additionally, the maps show that Fe is homogeneously incorporated in the catalytic layer, around 1% in the case of Ni_2_P and 0.7% in the case of Ni_3_S_2_.

Although the post-mortem XPS analysis showed similar structural composition for the hydroxides generated from different Ni-Xide precatalysts in P-KOH and Un-KOH, the geometric as well as the intrinsic catalytic activities are contrastingly different. Operando Raman spectroscopy was employed to gain further insights into the catalyst transformation mechanisms in real time via monitoring of the evolved surface species as a function of applied potential. Of note, the Raman spectra were collected for freshly activated samples after reaching a stable electrochemical response to align with the electrochemical data introduced earlier in this section. Figure 6 shows the Raman signals for Ni-hydroxide generated from Ni_2_P in P- and Un-KOH. The same set of data for Ni_3_S_2_ and Ni_2_B are introduced in Figures S8 and S9, respectively. In P-KOH, at low potential (1.03 V vs RHE) a broad band (ca. 450–500 cm^–1^) attributed to Ni–O and Ni–OH vibrations in Ni(OH)_2_ can be observed, which remains observable up to a potential of 1.23 V vs RHE.^41,42^ At 1.33 V vs RHE, the center of the band started to shift toward higher energy (ca. 480 cm^–1^) while another small band started to emerge at a higher energy of ca. 560 cm^–1^. With increasing potential, the Ni^2+^/Ni^3+^ oxidation potential or higher, the bands at 480 and 560 cm^–1^ intensified and became more resolved.^42^ These two bands are attributed to Ni–O bending and stretching vibrations in NiOOH and thus their intensification with the potential indicates phase transformation from Ni(OH)_2_ to NiOOH, the actual active form of the OER catalyst, regardless of the starting phase. The Raman response in Un-KOH (Figure 6b) is generally the same as the one in P-KOH except that the signals in P-KOH are stronger and the peaks are sharper in relation to those in Un-KOH even though the initial loading of the catalytic materials was the same and they were subjected to the same electrochemical conditions. This gives a further strong hint that more significant reconstruction happened in P-KOH, thus aligning with the previous discussion.^42^ It is well established that NiOOH can exist in two phases, namely, γ-NiOOH and β-NiOOH, which exhibit different Raman responses. Generally, β-NiOOH shows a higher stretching/bending (*I*_s_/*I*_b_) vibration ratio than the γ-NiOOH phase.^41,43^

In the current case, the signals at 560 cm^–1^ are way higher than the one at 480 cm^–1^ in both P-KOH and Un-KOH, indicating a predominant β-NiOOH phase as the catalytic structure rather than γ-NiOOH. The same holds true for Ni_3_S_2_ and Ni_2_B (Figures S11 and S12), which corroborates the previous XPS data that showed that all the materials transformed to similar hydroxides regardless of the original structure. Of interest at this point is the possibility to use the collected operando Raman data to understand the difference in catalytic behaviors for the set of Ni-Xides studied in this work. To do so, the *I*_s_/*I*_b_ values for all the three materials in P-KOH and Un-KOH at 1.53 V vs RHE, that is under OER conditions, are extracted and compared in Figure 6c. In P-KOH, Ni_3_S_2_ showed the highest *I*_s_/*I*_b_ ratio (1.93), meaning a higher extent of β-NiOOH relative to the other two cases, where Ni_2_P showed the lowest value (1.88). In Un-KOH, the *I*_s_/*I*_b_ values follow the following order: Ni_3_S_2_ > Ni_2_P > Ni_2_B, yet the general ratios support that β-NiOOH is still the predominant phase. What is interesting at this point is the drop in *I*_s_/*I*_b_ values between P- and Un-KOH for each sample, which relates to the transformation of β-NiOOH to γ-NiOOH induced by the presence of Fe.^41^ This transformation is considered a kind of disorder in the NiOOH structure and is associated with inducing higher catalytic activity. The drop in the *I*_s_/*I*_b_ ratio (the extent of disorder) is 7% in the case of Ni_2_B while it is around 4% in the other two cases (Ni_2_P and Ni_3_S_2_). Accordingly, NiOOH derived from Ni_2_B showed the highest disorder that correlates with the highest intrinsic catalytic activity among the samples.

## Conclusions

In this study, we examined the surface reconstruction of Ni-Xides (X = B, P and S) and the impact on the properties of *in situ*-derived nickel hydroxides and the OER electrocatalytic activity. By studying three materials, namely, Ni_2_P, Ni_3_S_2_, and Ni_2_B, possessing a close Ni/X ratio but with different X atomic radii and electronegativity, in purified KOH (P-KOH) and unpurified KOH (Un-KOH) to decouple the role of possible Fe incorporation, we were able to derive structure–activity correlations. Electrochemical analysis showed that the three materials exhibit markedly higher activity in Un-KOH but much lower and similar activity if they were tested in P-KOH, pointing to the decisive role that Fe inclusion plays on the OER activity of Ni-Xide precatalysts, an overlooked factor in the literature. Online ICP-OES analysis showed a significantly higher extent of P and S dissolution in P-KOH, implying a higher extent of reconstruction, however, the OER activity was contrastingly much lower compared to measurements in Un-KOH. Reconstruction is thus evidently not the main activity driver. Data on B dissolution was not included due to experimental challenges with its reliable quantification using the ICP-OES. In contrast, both Ni_2_P and Ni_3_S_2_ showed apparently higher OER activity in Un-KOH compared to Ni_2_B based on current normalization with respect to electrode geometric area, however, Ni_2_B exhibited a higher intrinsic activity. This can be rationalized based on the smaller thickness of the *in situ*-derived hydroxide layer inferred from the Ni^2+^/Ni^3+^ charge integration and a smaller double layer capacitance (*C*_dl_), which might induce faster electron transport across the hydroxide/boride junction, as reflected also in the smaller Tafel slope of Ni_2_B (41.33 mV dec^–1^) relative to Ni_2_P (44.80 mV dec^–1^) and Ni_3_S_2_ (46.29 mV dec^–1^). Additionally, the fact that the Ni_2_B core is expected to be more conductive compared to Ni_2_P and Ni_3_S_2_ owing to the lower electronegativity of B relative to both P and S could favor the formation of a thinner hydroxide layer that facilitates a faster electron transport path. *In situ* Raman spectroscopy disclosed that the hydroxide derived from the reconstruction of Ni_2_B possesses a higher extent of disorder than the other two cases, thus corroborating the fact that the higher intrinsic activity of Ni_2_B originates from the unique properties of its hydroxide structure in alignment with the electrochemical analysis.

## Experimental Section

### Synthesis of Ni_2_P

Ni_2_P was synthesized via phosphorization of presynthesized Ni(OH)_2_. In a typical synthesis, 4.2 mmol of NiCl_2_·6H_2_O was dissolved in 20 mL of DI water to form solution A. In another beaker, 0.2732 g of NaOH and 0.2258 g of Na_2_CO_3_ were dissolved in 20 mL of DI water forming solution B. Both solutions A and B were added dropwise to a round bottomed flask containing 40 mL of DI H_2_O under magnetic stirring, while keeping the ratio of A:B at 2:1. The rest of solution B was used to adjust the reaction mixture to pH 8.5.^44^ The reaction mixture was kept under stirring for 24 h. The precipitate was then collected by centrifugation, washed with DI water and ethanol successively, and dried overnight at 70 °C. Then, 46 mg of the green powder was loaded in a ceramic boat separately in the upstream position from 315 mg of sodium hypophosphite powder. The boat was positioned in the middle of a tube furnace and heated to 300 °C in 1 h ramping time and held for 1.5 h under an Ar atmosphere. The obtained black powder was finely ground and used without further purification.

### Synthesis of Ni_3_S_2_

Ni_3_S_2_ was synthesized via a microwave-assisted solvothermal method adapted from Sun et al.^35^ with few modifications. In a typical synthesis, 1 mmol of Ni(CH_3_COO)_2_·4H_2_O was dissolved in 10 mL of oleylamine under stirring until complete dissolution in a 35 mL Anton Paar glass vial. Subsequently, 0.6 mmol of elemental S was introduced followed by stirring for 30 min. The reaction mixture was heated up to 260 °C in 5 min and held for 10 min in an Anton Paar 350 Microwave under stirring, followed by cooling down to room temperature gradually. The product was collected and washed three times with ethanol and dried under air at 50 °C. The obtained black powder was finely ground and stored in a desiccator under an argon atmosphere for further use.

### Synthesis of Ni_2_B

Ni_2_B was synthesized using a molten-salt-assisted boronization method adapted from Guo et al.^36^ with modifications. In a typical synthesis, 115 mg (0.5 mmol) of NiCl_2_·6H_2_O was ground with 8.25 mg (0.75 mmol) of elemental B in a mortar using a pestle for 5 min to form mixture A. Next, 0.705 g of KCl and 0.577 g of LiCl were mixed for 5 min to form mixture B. A and B were ground together for 5 min to form a homogeneous reaction mixture. This mixture was loaded in a ceramic boat and introduced into a tube furnace. After Ar purging for 30 min, the temperature was raised to 750 °C (10 °C/min) and held for 1.5 h followed by natural cooling to room temperature. The mixture was washed with hot DI water and absolute ethanol several times to get rid of the reaction byproducts followed by drying at 60 °C for 5 h.

### Electrochemical Characterization

The electrochemical measurements were conducted in a three-electrode configuration with a BioLogic, VSP equipped with EIS in a homemade electrochemical cell constructed from polyether ether ketone (PEEK). Hg/HgO (1.0 M KOH) and Pt wire were employed as the reference and counter electrode, respectively. The catalyst ink was prepared by dispersing 1.5 mg of the catalyst powder in a 1000 μL solution of DI water:ethanol:5% Nafion binder (485:485:30 μL) followed by 30 min sonication to form a homogeneous ink. Four microliters of the ink was drop cast on a precleaned GC tip (0.196 cm^–2^) to reach a loading of 30 μg/cm^–2^. The glassy carbon tip was inserted into a Teflon holder as an RDE tip controlled by a rotator (Autolab, Metrohm, Switzerland). Electrochemical measurements were conducted in 1.0 M P/Un-KOH at 1600 rpm. The KOH solution was purified electrochemically by applying a constant current of 0.2 A in a two electrode setup for 24 h using a NiS_3_–MoS_2_ catalyst deposited on Ni foam according to Spanos et al., where the Fe concentration was monitored with ICP-OES.^29^ The catalysts were cycled for 50 CVs in the potential window 0.1–0.75 V vs Hg/HgO (1.0 M KOH) at a scan rate of 20 mV s^–1^, followed by 2 CVs in the potential window 0.1–0.8 V vs Hg/HgO (1.0 M KOH) at a scan rate of 5 mV s^–1^. EIS measurements were performed over a frequency range from 100 kHz to 1.0 Hz at 0.75 V vs HgO using an AC bias potential of 10 mV. The EIS data were used to extract the double layer capacitance (*C*_dl_) using the one-time constant equivalent circuit model shown in the inset of Figure S5b, and the respective calculations are described. A 100% IR correction is used for all presented measurements. The potentials were corrected to the RHE scale according to the Nernst equation [*E*_RHE_ = *E*_Hg/HgO_ + 0.059 × pH + *E*°_Hg/HgO_], where *E*°_Hg/HgO_ in 1.0 M KOH concentration at 25 °C is 0.098 V.

### Online Dissolution Studies by ICP-OES

Online ICP-OES was conducted to determine the dissolution rate of the guest elements (P and S) during the OER in 0.1 M P-KOH and Un-KOH. An electrochemical flow cell with a glassy carbon working electrode area of 0.196 cm^2^ coupled with an ICP-OES (Spectroblue EOP, Ametek) was employed. The same catalyst loading was used as in the RDE measurements. As with the previous activation, 50 CVs at a rate of 20 mV s^–1^ were recorded to study the dissolution rate. The electrolyte stream was injected at a flow rate of 0.86 mL/min in a quartz nebulizer operated at an Ar (99.999% purity) flow rate of 0.86 L/min. A background signal of P and S was recorded for 5 min before and after the electrochemical measurements at open-circuit voltage to establish an appropriate baseline that was subtracted from the data collected during CV measurements.

### *In Situ* Raman Spectroscopy

Raman measurements were conducted with a commercial *in situ* Raman cell purchased from redox.me coupled with Oceanview Raman spectroscopy. The catalysts were loaded on preroughened gold tips to enhance the signal-to-noise ratio. These catalysts were preconditioned for 50 CVs in either P-KOH or Un-KOH then used directly in the cell to conduct measurements in the same solution. Data was collected by applying potential steps of 1.03, 1.13, 1.23, 1.33, 1.38, 1.43, 1.48, and 1.53 V vs RHE with a holding time of 20 s per step to ensure signal stabilization.

### Catalysts Characterization

XRD measurements were conducted on a benchtop D Phaser diffractometer (Bruker) using a Cu Kα radiation source (λ = 0.154184 nm). HRTEM and HAADF-STEM images and EELS maps were acquired with a field emission gun FEI Tecnai F20 microscope operated at 200 kV. The HAADF-STEM EDS maps were acquired in a double corrected and monochromated Thermo Fisher Spectra 300 microscope operated at 60 kV with a probe convergence angle of 21.4 mrad.

XPS was carried out using a Kratos AXIS nova instrument with a monochromatic Al Kα X-ray source (pass energy of 1486.6 eV and an emission current of 15 mA). For all measurements, a low-energy electron flood was applied for charge neutralization. The binding energy scale of the measurement data was calibrated based on an assignment of the C 1s peak component of adventitious carbon to an energy of 284.8 eV.

## Supporting Information Available

Pre- and post-electrocatalysis characterization data (XRD, SEM, and TEM), electrochemical results, and operando Raman spectroscopy data (PDF)

## Abbreviations

OER: oxygen evolution reaction
Un-KOH: unpurified KOH
P-KOH: purified KOH
EWS: electrochemical water splitting
HER: hydrogen evolution reaction
ECSA: electrochemical active surface area
CV: cyclic voltammetry
*C*_dl_: double layer capacitance
XPS: X-ray photoelectron spectroscopy
ICP-OES: inductively coupled plasma optical emission spectroscopy
