# Supplementary material for: Ni-Xides (B, S, and P) for Alkaline OER: Shedding Light on Reconstruction Processes and Interplay with Incidental Fe Impurities as Synergistic Activity Drivers
Source: ACS Appl Energy Mater. 2024 Feb 8;7(4):1369–81. doi: 10.1021/acsaem.3c03114 (PMC10900598; doi:10.1021/acsaem.3c03114)
Supplement: Supplementary file 1 — ae3c03114_si_001.pdf [file ae3c03114_si_001.pdf]

Supporting Information

Ni-Xides (B, S, and P) for alkaline OER: Shedding light on reconstruction processes and inter-play with incidental Fe impurities as synergistic activity drivers

Sayed Mahmoud El-refaei, David Llorens Rauret, Alba G. Manjón, Ioannis Spanos, Aleksandar Zeradjanin, Stefan Dieckhöfer, Jordi Arbiol, Wolfgang Schuhmann and Justus Masa**

Sayed M. El-refaei- Max-Planck-Institut für Chemische Energiekonversion, Stiftstraße 34-36, 45470 Mülheim an der Ruhr, Germany. Corresponding author: Sayed.Elrefaei@cec.mpg.de

Alba G. Manjón- Institut Català de Nanociència i Nanotecnologia (ICN2), CSIC &BIST, Spain

David L. Rauret- Institut Català de Nanociència i Nanotecnologia (ICN2), CSIC &BIST, Spain

Stefan Dieckhöfer- Analytical Chemistry, Center for Electrochemical Sciences (CES), Faculty of Chemistry and Biochemistry, Ruhr University Bochum, Universitätsstr. 150, D-44780 Bochum, Germany

Jordi Arbiol- Institut Català de Nanociència i Nanotecnologia (ICN2), CSIC &BIST, Spain

Ioannis Spanos- Max-Planck-Institut für Chemische Energiekonversion, Stiftstraße 34-36, 45470 Mülheim an der Ruhr, Germany

Aleksandar Zeradjanin- Max-Planck-Institut für Chemische Energiekonversion, Stiftstraße 34-36, 45470 Mülheim an der Ruhr, Germany

Wolfgang Schuman- Analytical Chemistry, Center for Electrochemical Sciences (CES), Faculty of Chemistry and Biochemistry, Ruhr University Bochum, Universitätsstr. 150, D-44780 Bochum, Germany

Justus Masa- Max-Planck-Institut für Chemische Energiekonversion, Stiftstraße 34-36, 45470 Mülheim an der Ruhr, Germany. **Corresponding author**: Justus.Masa@cec.mpg.de

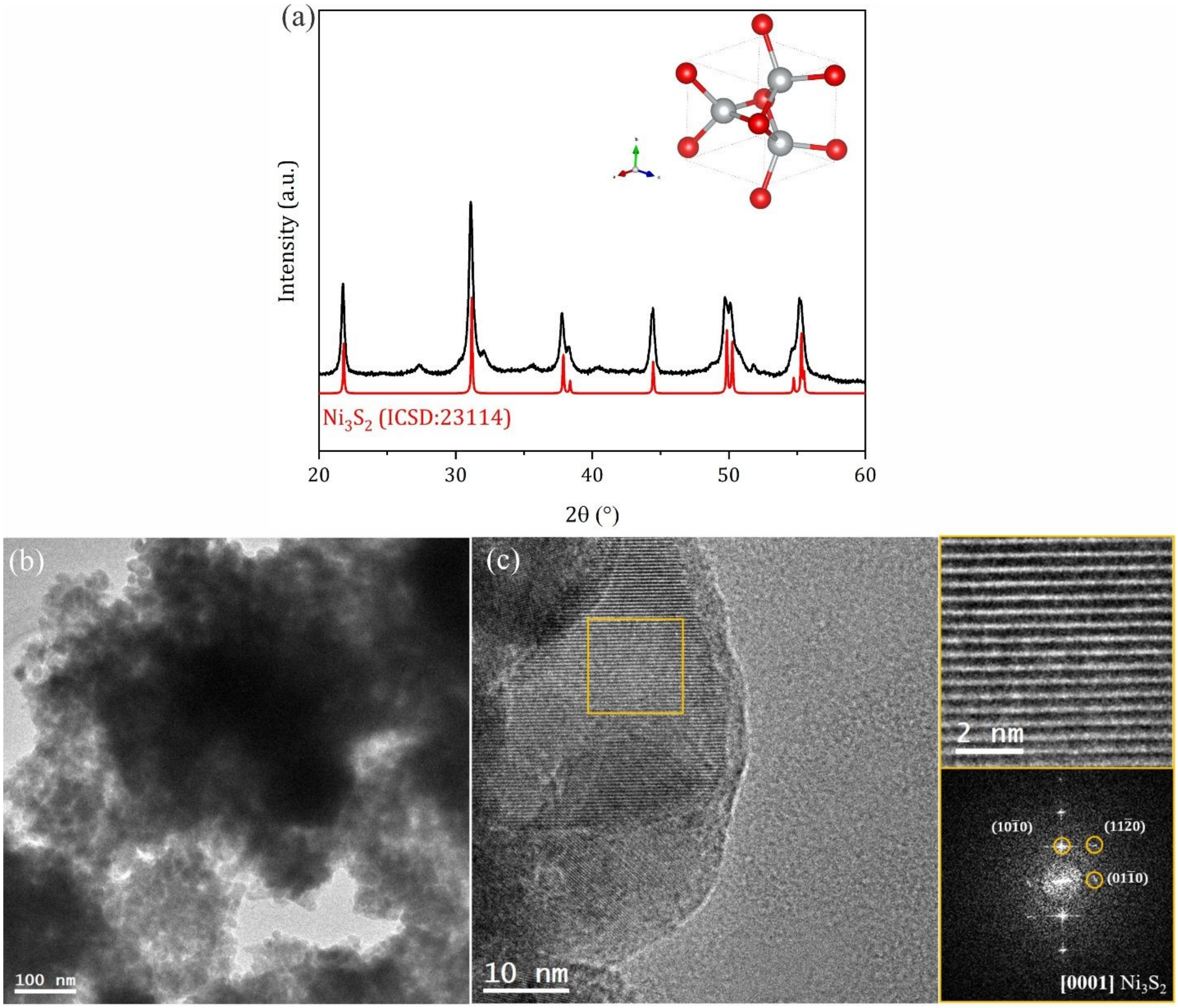

Figure S1. (a) XRD of Ni_3S_2. (b) TEM, (c) HR-TEM with corresponding FFT analysis.

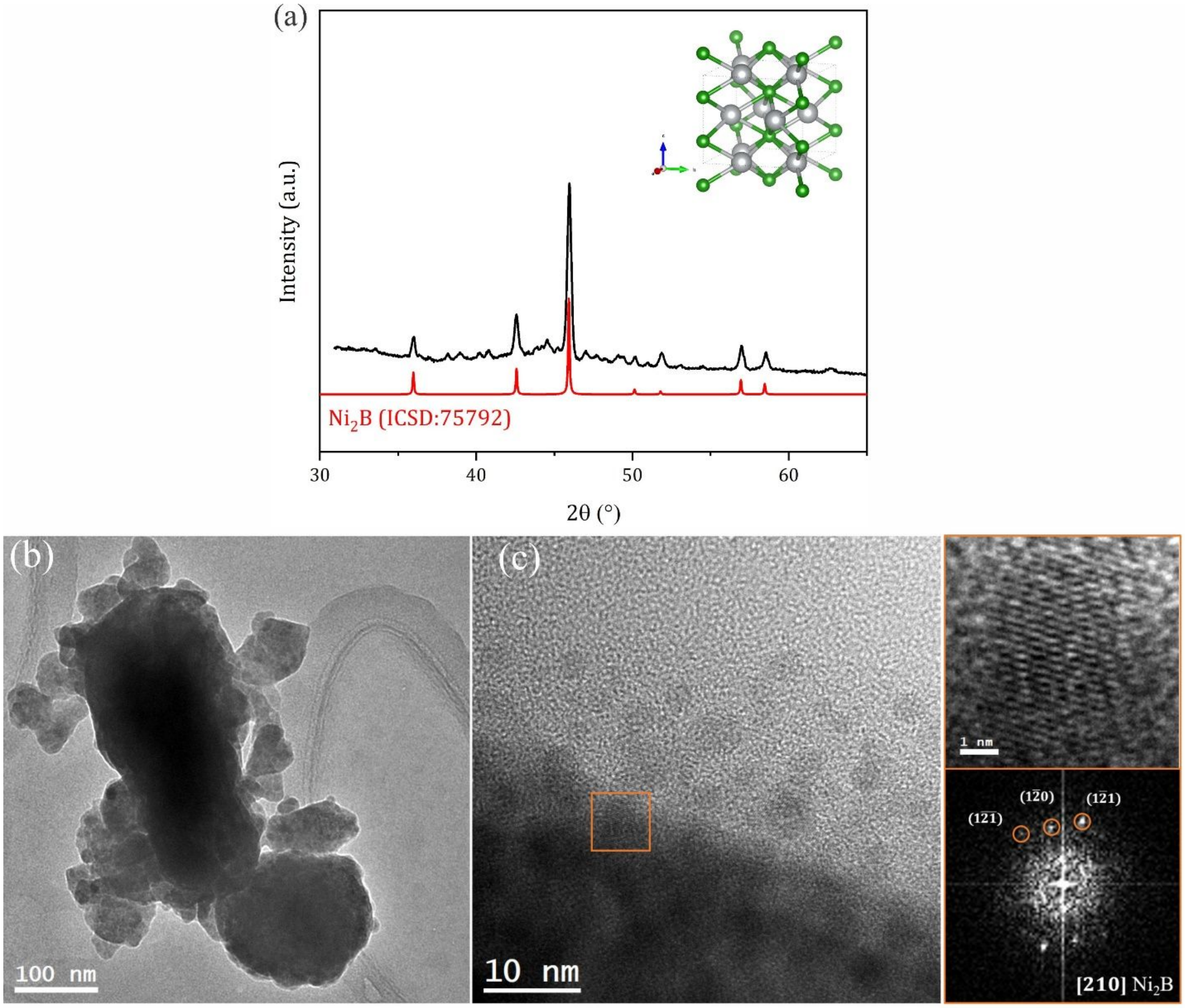

Figure S2. (a) XRD of Ni_2B. (b) TEM, (c) HR-TEM with corresponding FFT analysis.

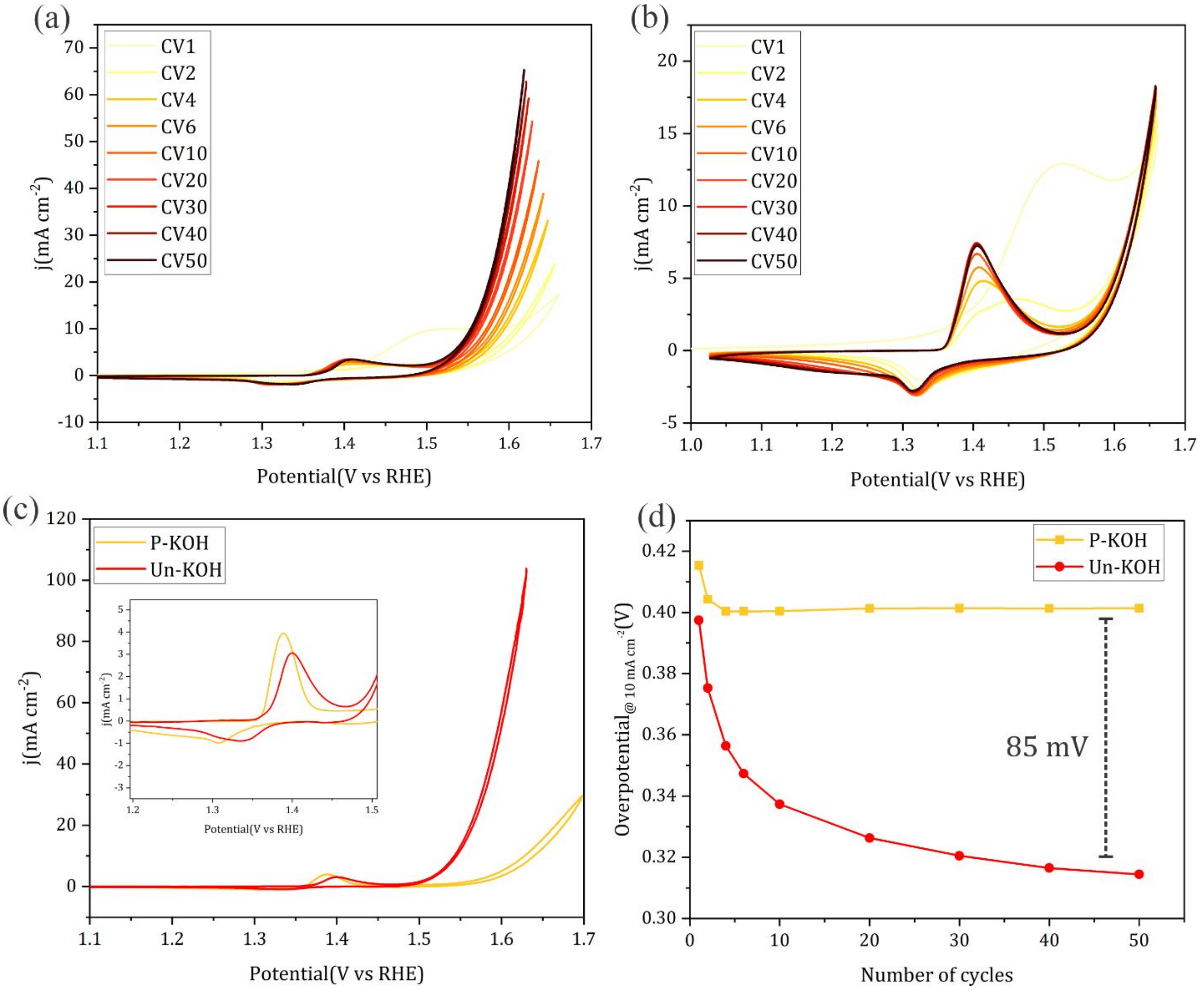

Figure S3. Continues CVs of Ni_3S_2 at 20 mV s^{-1} in 1.0 M KOH (a) unpurified and (b) purified. (c) Comparison CVs of OER performance at 5.0 mV s^{-1} after 50 CVs (a and b), inset is the magnification of $Ni^{2+}/^{3+}$ redox peaks. (d) The corresponding overpotentials at 10 mA cm^{-2} derived from a and b.

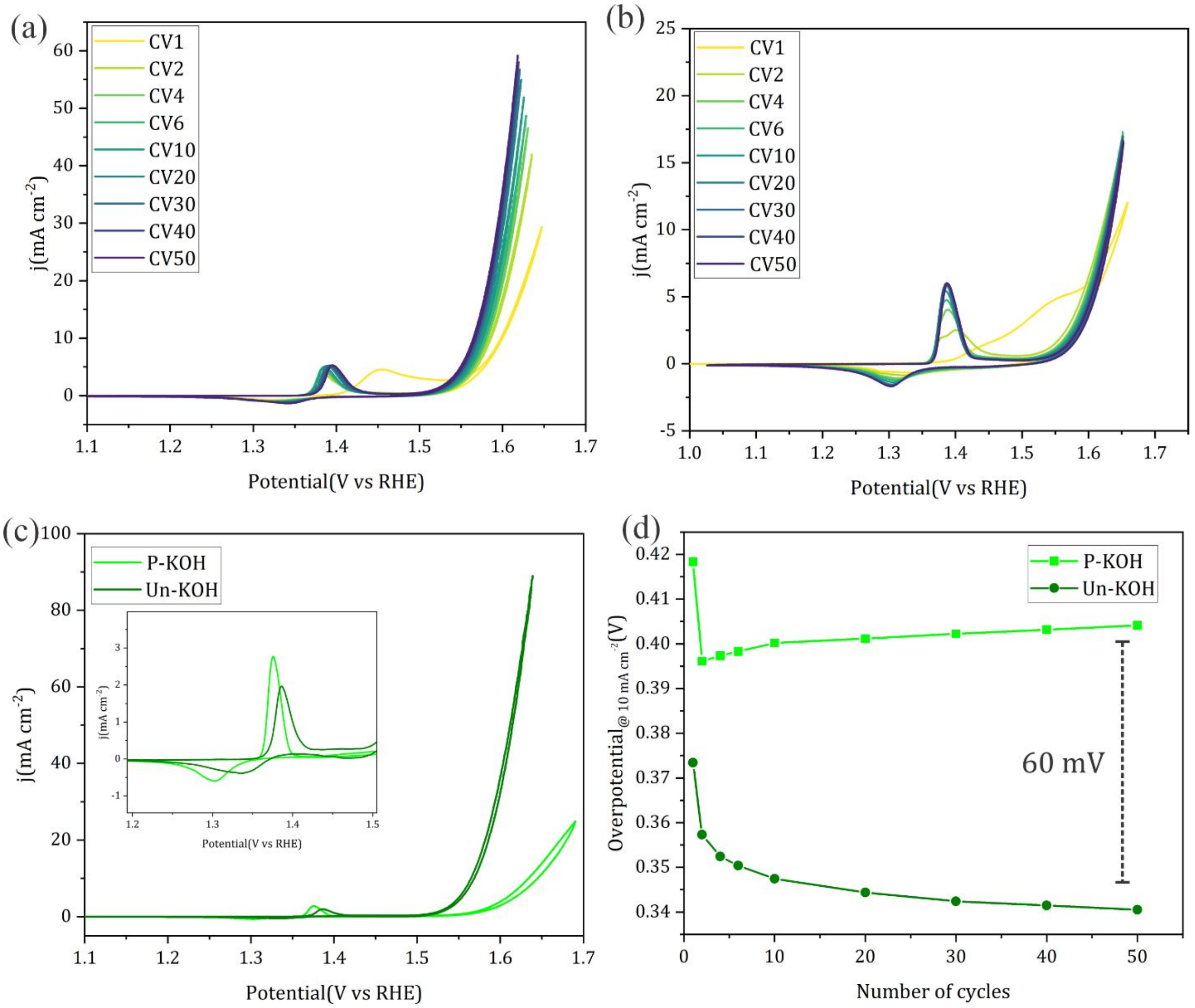

Figure S4. Continues CVs of Ni2B at 20 mV s^{-1} in 1.0 M KOH (a) unpurified and (b) purified. (c) Comparison CVs of OER performance at 5.0 mV s^{-1} after 50 CVs (a and b), inset is the magnification of $Ni^{2+}/^{3+}$ redox peaks. (d) The corresponding overpotentials at 10 mA cm^{-2} derived from a and b.

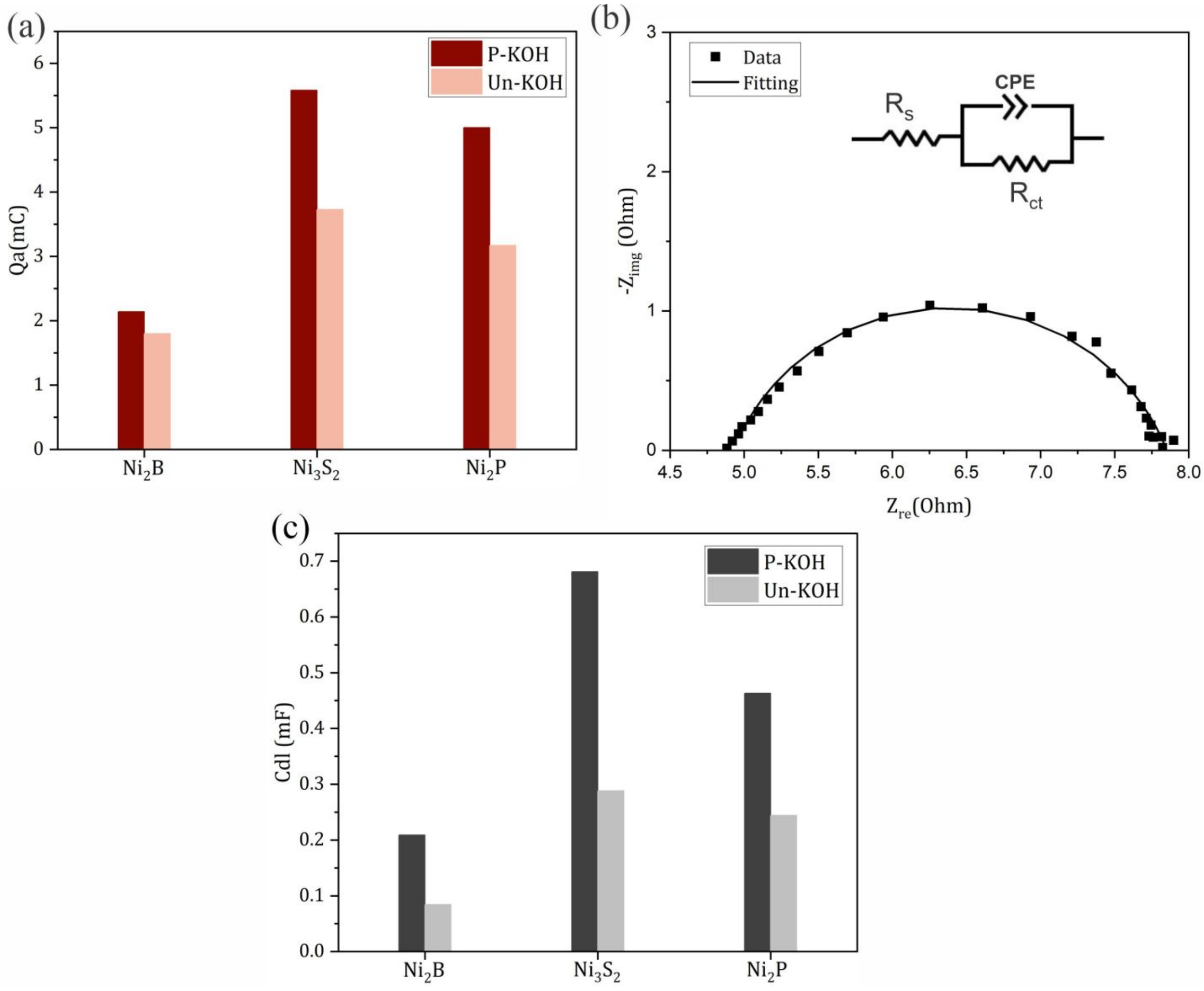

Figure S5. (a) Anodic peak redox charge integrated after 50 CVs. (b) EIS of Ni_2P in 1.0 UN-KOH (inset is the fitting model discussed below). (c) C_{dl} extracted from EIS measurements at 1.67 V RHE in 1.0 M unpurified and purified KOH.

All measured EIS data in P-KOH and Un-KOH were fitted with the model introduced in Figure 5b inset. The values of the C_{dl} (mF) were calculated using the following equation,

$$C_{dl} = \left(\frac{CPE}{((R_s)^{-1} + (R_{ct})^{-1})^{1-n}}\right)^{\frac{1}{n}}$$

where Rs (ohm) is the solution resistance, R_{ct} (Ohm) is the charge transfer resistance, CPE (F s^{1-n}) is the constant phase element, and n is the power of CPE.[1]

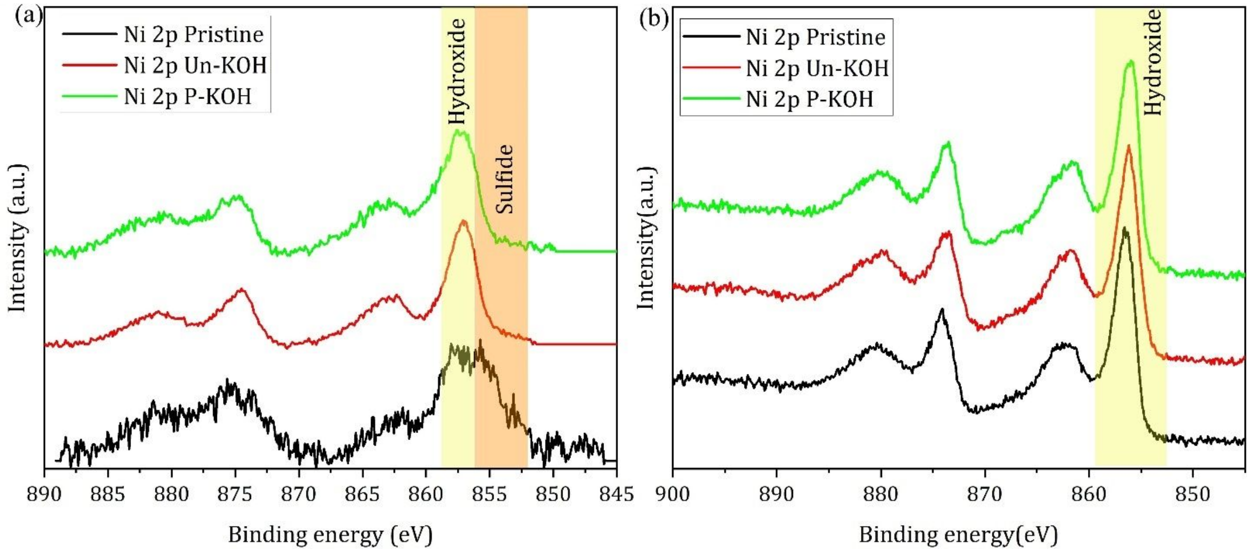

Figure S6. XPS spectra of Ni 2p core level for (a) Ni_3S_2, and (b) Ni2B for the pristine and reconstructed samples for 50 CVs in Un-KOH and P-KOH.

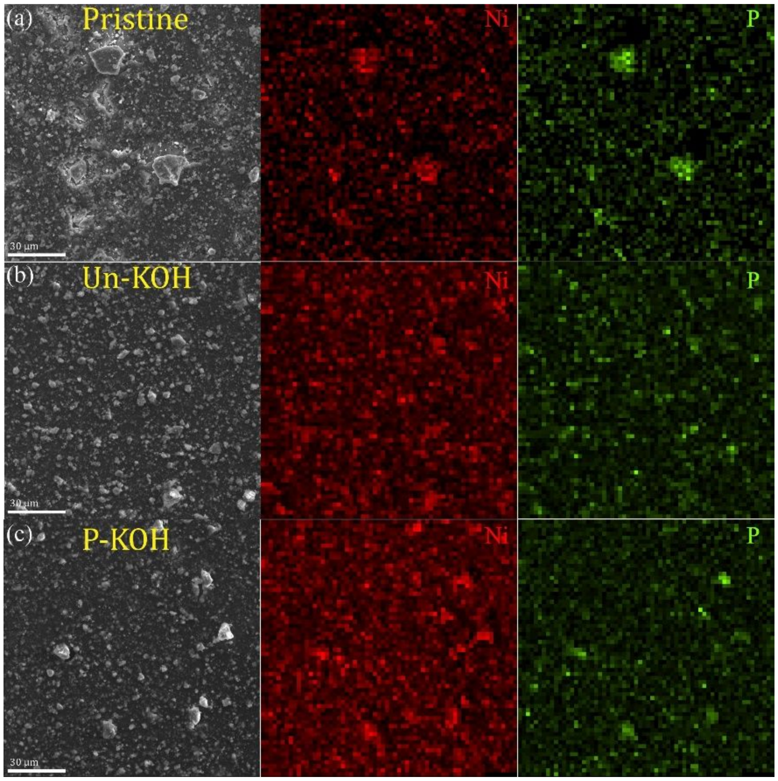

Figure S7. SEM and corresponding EDS mapping of Ni_2P coated GC electrode for (a) pristine, (b) in Un-KOH and (c) P-KOH.

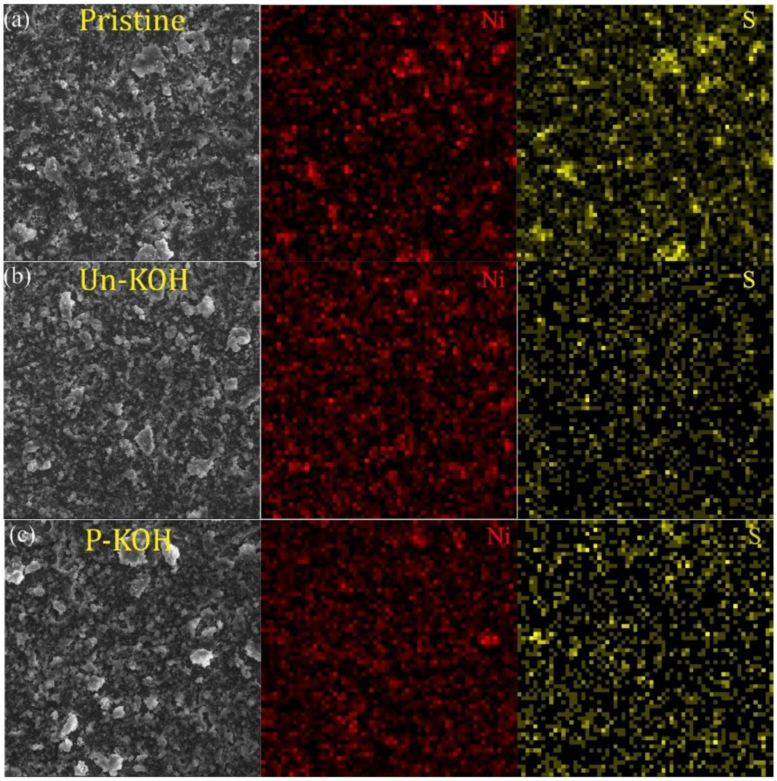

Figure S8. SEM and corresponding EDS mapping of Ni_3S_2 coated GC electrode for (a) pristine, (b) in Un-KOH and (c) P-KOH.

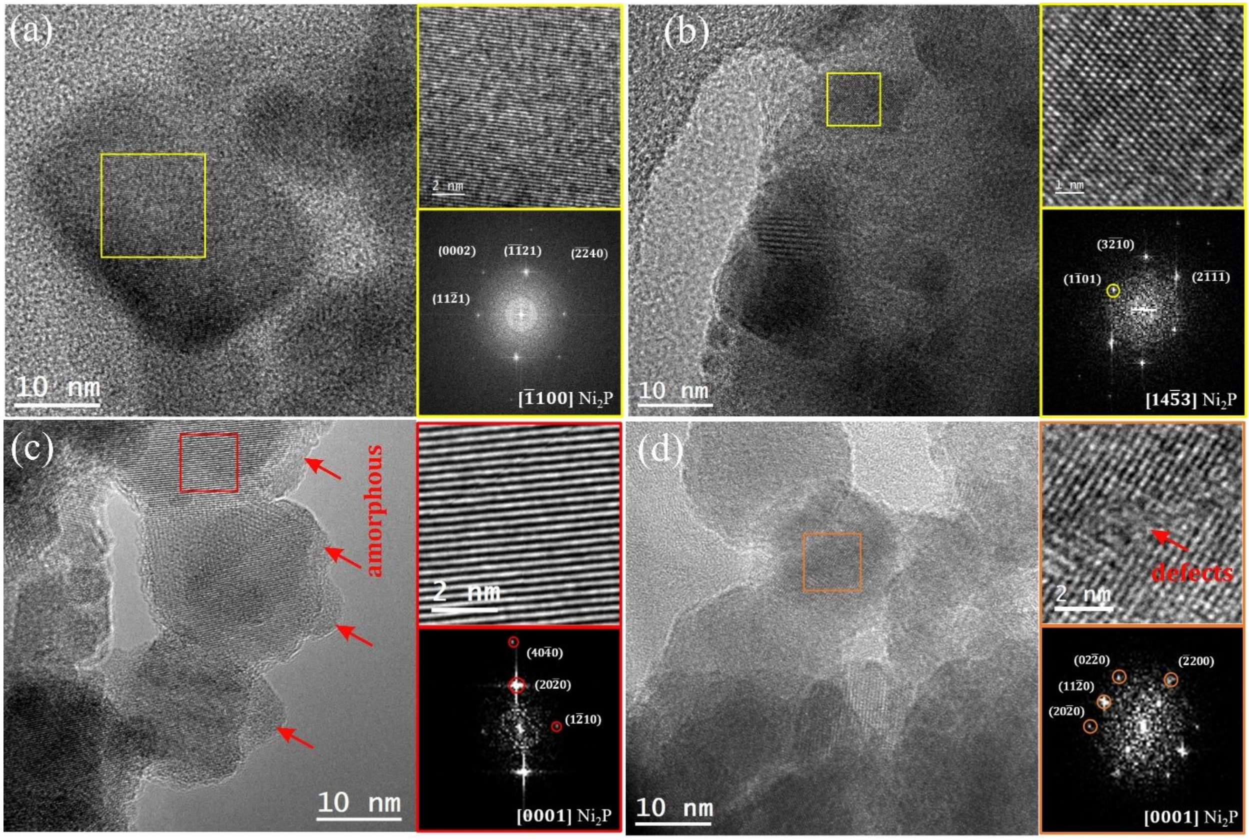

Figure S9. TEM/HR-TEM and selected area FFT analysis for Ni_2P after activation of 50 CVs in (a, b) Un-KOH and (c, d) P-KOH.

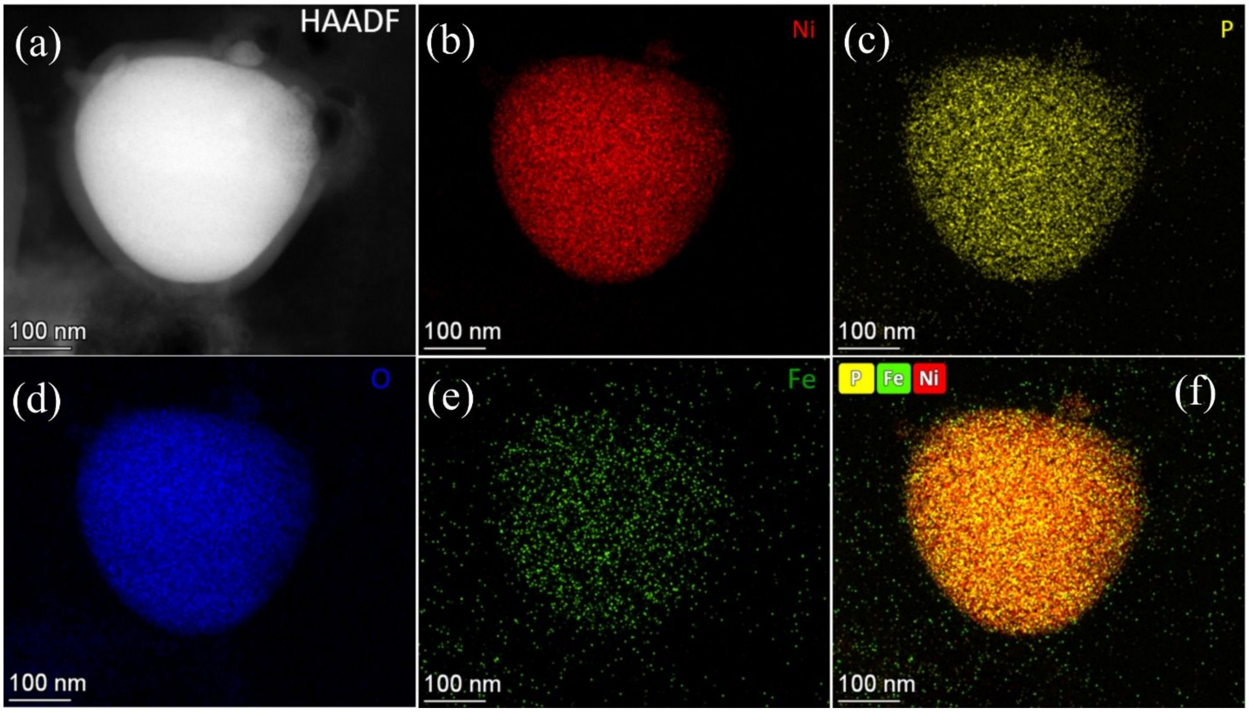

Figure S10. (a) HAADF and (b-f) EDS mapping of Ni_2P after activation for 50 CVs in Un-KOH.

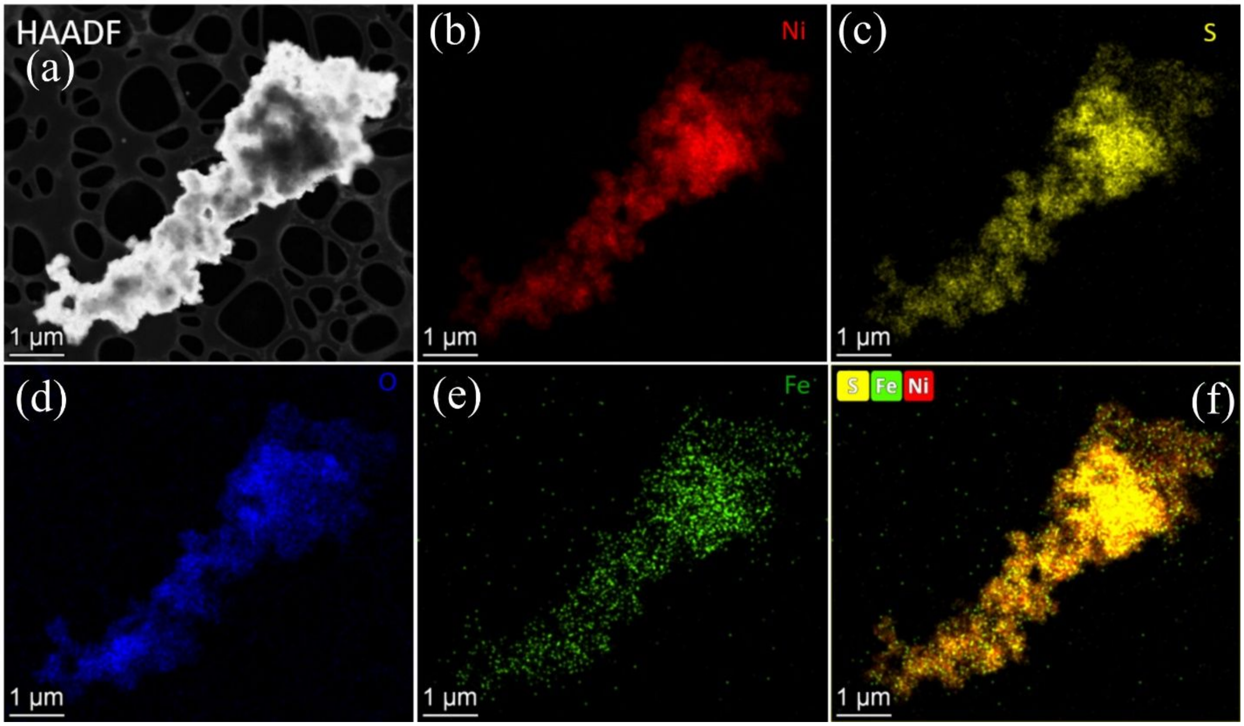

Figure S11. (a) HAADF and (b-f) EDS mapping of Ni_3S_2 after activation for 50 CVs in Un-KOH.

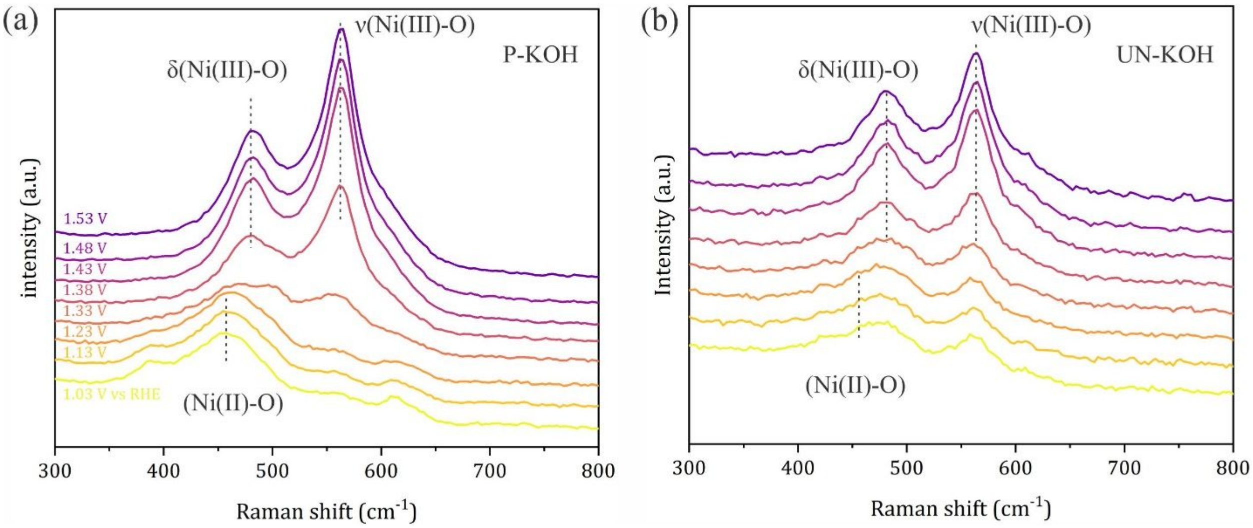

Figure S11. Raman spectra of activated Ni_3S_2 at different potentials in (a) P-KOH and (b) Un-KOH.

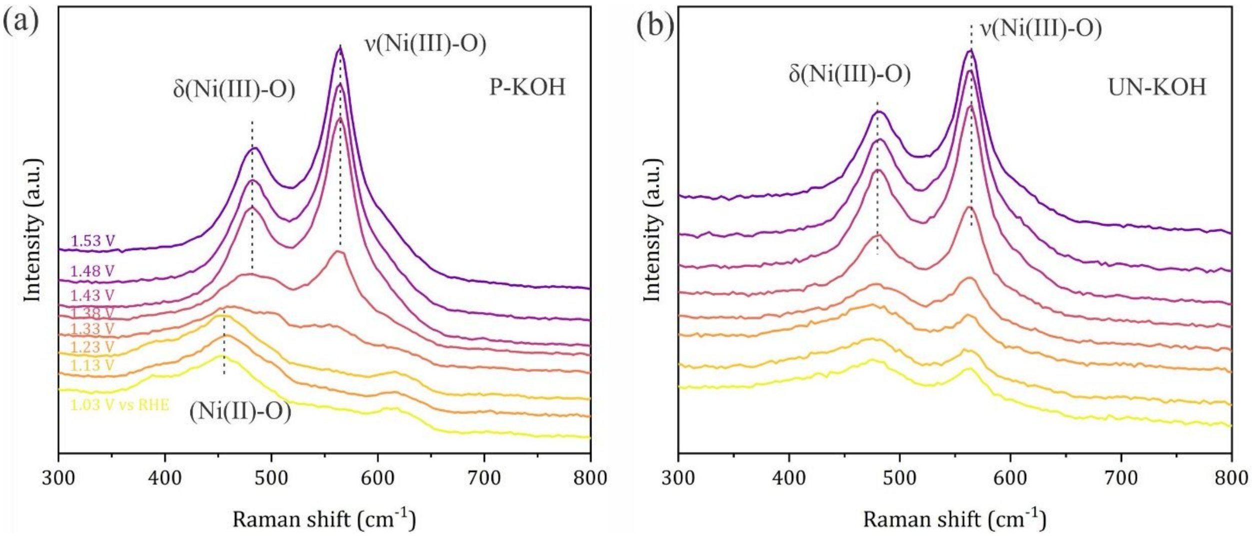

Figure S12. Raman spectra of activated Ni_2B at different potentials in (a) P-KOH and (b) Un-KOH.

References

(1) Bao, F.; Kemppainen, E.; Dorbandt, I.; Xi, F.; Bors, R.; Maticiuc, N.; Wenisch, R.; Bagacki, R.; Schary, C.; Michalczik, U.; et al. Host, Suppressor, and Promoter—The Roles of Ni and Fe on Oxygen Evolution Reaction Activity and Stability of NiFe Alloy Thin Films in Alkaline Media. *ACS Catal.* **2021**, *11* (16), 10537-10552. DOI: 10.1021/acscatal.1c01190.
